# Targeting Tumor Stroma: Current Challenges and Future Directions

**DOI:** 10.1002/mco2.70763

**Published:** 2026-05-10

**Authors:** Siwei Wang, Haofan Hu, Weifeng Zeng, Lu Qin, Xiaoping Chen, Zhibin Liao, Furong Liu, Zhanguo Zhang

**Affiliations:** ^1^ Division of Hepato‐Pancreato‐Biliary Surgery Tongji Hospital Tongji Medical College Huazhong University of Science and Technology Wuhan Hubei China; ^2^ Hubei Key Laboratory of Hepato‐Pancreato‐Biliary Diseases Wuhan Hubei China; ^3^ Key Laboratory of Organ Transplantation Ministry of Education NHC Key Laboratory of Organ Transplantation Key Laboratory of Organ Transplantation Chinese Academy of Medical Sciences Wuhan China; ^4^ Department of Anesthesiology Union Hospital Tongji Medical College Huazhong University of Science and Technology Wuhan China; ^5^ Key Laboratory of Anesthesiology and Resuscitation Huazhong University of Science and Technology Ministry of Education Wuhan Hubei China

**Keywords:** cancer‐associated fibroblasts, cancer therapy, clinical trial, extracellular matrix, targeted therapy, tumor stroma

## Abstract

Tumor stroma is a critical component of the tumor microenvironment (TME) that plays a pivotal role in cancer progression and therapeutic response. Beyond providing structural support, stromal components actively regulate tumor growth, metastasis, immune evasion, and drug resistance. Tumor stroma consists of both noncellular elements, particularly the extracellular matrix (ECM), and diverse stromal cells such as cancer‐associated fibroblasts and mesenchymal stem cells. These components collectively form a dense and dynamic barrier that restricts drug penetration, increases interstitial pressure, and promotes an immunosuppressive microenvironment. Despite growing recognition of the importance of stromal biology, a systematic understanding of stromal‐targeted therapeutic strategies and their translational potential remains incomplete. In this review, we comprehensively summarize the biological functions of major stromal components in tumor development and therapy resistance. We further discuss current therapeutic strategies targeting the tumor stroma, including stromal cell depletion, vascular normalization, ECM‐modulating approaches, and emerging nanomaterial‐based delivery systems designed to enhance drug penetration and therapeutic efficacy. In addition, recent progress in preclinical studies and clinical trials of stromal‐targeted therapies is highlighted. By integrating advances in tumor biology, nanomedicine, and translational oncology, this review provides a comprehensive perspective on stroma‐targeted cancer therapy and outlines future directions for precision medicine.

## Graphical abstract

Figure 1. Mechanism of ECM reprogramming. Cancer‐associated fibroblasts (CAFs), cancer‐associated macrophages (CAMs), and other mesenchymal cells alter the ECM composition and increase its stiffness by depositing matrix components such as collagen and hyaluronic acid, and secreting cross‐linking agents like lysyl oxidase homolog 2 (LOXL2). The altered composition and mechanics of the ECM amplify mechanical signals to cancer cells, thereby regulating downstream factors such as the zinc finger protein SNAI1 (Snail) and hypoxia‐inducible factor 1α (HIF1α). These changes promote tumor progression by enhancing cell proliferation, invasion, survival, and treatment resistance.

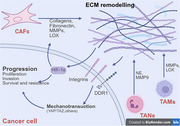

## 1 Introduction

The tumor stroma, once regarded as a passive scaffold merely providing structural support for neoplastic cells, has been radically redefined over the past two decades. Accumulating evidence now unequivocally positions it as an active and indispensable participant in the tumor microenvironment (TME), playing pivotal roles in cancer initiation, progression, metastasis, and therapeutic resistance [1, 2, 3, 4, 5, 6]. This paradigm shift stems from the recognition that stromal components—including the extracellular matrix (ECM) and a diverse array of cells such as cancer‐associated fibroblasts (CAFs), mesenchymal stem cells (MSCs), and vascular endothelial cells (ECs)—engage in dynamic, reciprocal crosstalk with tumor cells [6]. They collectively establish a dense, fibrotic, and immunosuppressive niche that not only fosters tumor survival but also erects formidable physical and biological barriers against conventional therapies, such as impeding drug penetration and facilitating immune evasion [7, 8, 9, 10]. Consequently, the tumor stroma has transformed from a traditional “bystander” into a compelling therapeutic target, giving rise to the innovative strategy of stroma‐targeted cancer therapy [11, 12].

Despite the growing recognition of stromal biology's importance and the proliferation of research in this field, a systematic and integrated understanding of stroma‐targeted therapeutic strategies and their translational potential remains incomplete. Numerous reviews have extensively detailed the biological functions of individual stromal components. However, there is a pressing need for a comprehensive synthesis that not only summarizes the roles of key stromal elements but also critically evaluates the current arsenal of therapeutic strategies—from stromal depletion and ECM modulation to vascular normalization—alongside the progress of targeting stromal elements in clinical trials. Furthermore, the emerging promise of nanomaterial‐based delivery systems designed to overcome stromal barriers and enhance therapeutic efficacy warrants a focused discussion. This review aims to bridge these gaps by providing a cohesive perspective on the translation of insights from stromal biology into clinical practice.

In this review, we first comprehensively dissect the biological functions of major stromal components within the TME, including the ECM and key stromal cell populations. We then methodically discuss current and emerging therapeutic strategies targeting the tumor stroma, encompassing direct targeting of ECM components (e.g., hyaluronan [HA], collagen) and their remodeling enzymes (e.g., lysyl oxidase (LOX), matrix metalloproteinase [MMPs]) [13, 14], interventions against specific stromal cells (e.g., CAFs, tumor‐associated macrophages (TAMs)) [15, 16], and approaches to normalize the aberrant tumor vasculature [17]. Importantly, we highlight recent progress in both preclinical studies and clinical trials of these stroma‐targeted therapies, providing a translational outlook. Finally, we explore the integration of nanomedicine in this context, reviewing advanced nanocarrier systems engineered to enhance drug delivery and penetration within the dense stromal matrix [18, 19, 20]. Overall, this article is organized to provide a coherent progression from stromal biology to therapeutic strategies and finally to emerging nanotechnology‐enabled approaches, followed by a discussion of current challenges and future directions in stroma‐targeted cancer therapy.

## 2 Role of Tumor Stroma in the TME

This section delves into the multifaceted biological functions of the three key components of the tumor stroma: the ECM, CAFs, and MSCs. We will first dissect how the remodeled ECM establishes physical and biochemical barriers that drive tumor stiffness, impede drug penetration, and facilitate immune evasion through intricate mechanotransduction signaling. Subsequently, we will explore the extensive heterogeneity and dual roles of CAFs, highlighting their capacity to both suppress and, in certain contexts, promote antitumor immunity by recruiting and training various immune cell populations. Finally, the discussion will turn to MSCs, detailing their protumorigenic effects—such as promoting angiogenesis, epithelial–mesenchymal transition (EMT), and fostering an immunosuppressive niche. Collectively, this section establishes a foundational understanding of why the tumor stroma represents a compelling therapeutic target.

### 2.1 Extracellular Matrix

**FIGURE 1:**
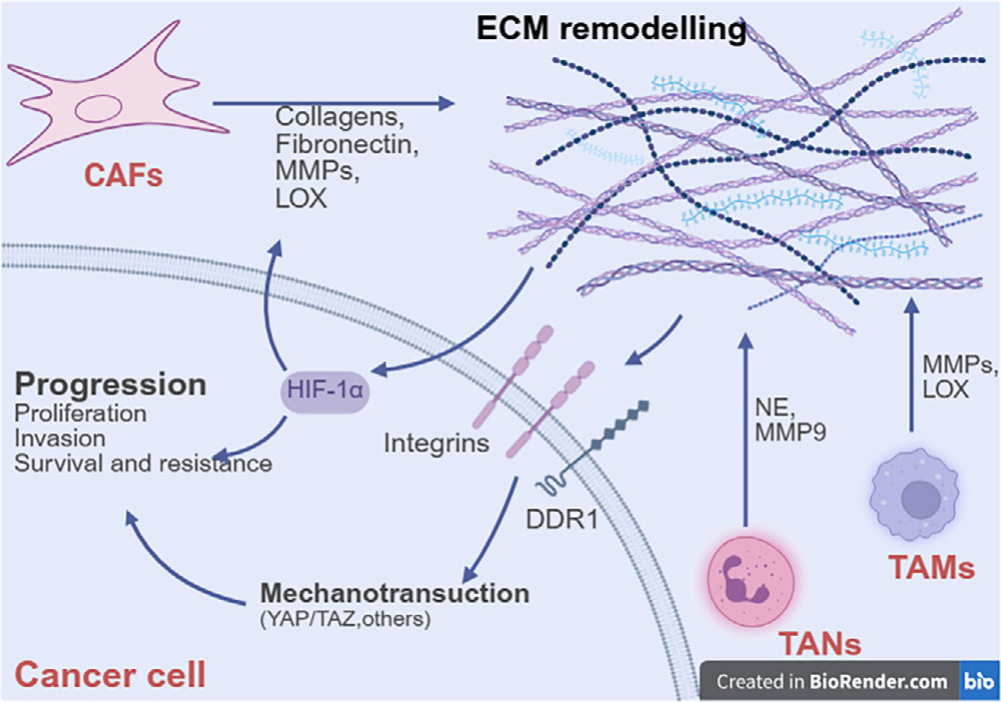
Mechanism of ECM reprogramming. Cancer‐associated fibroblasts (CAFs) and other mesenchymal cells directly alter the ECM composition and increase its stiffness by depositing matrix components such as collagen and hyaluronic acid, and by secreting cross‐linking enzymes like lysyl oxidase homolog 2 (LOXL2). In contrast, tumor‐associated macrophages (TAMs) primarily act as regulators that indirectly influence ECM remodeling through the secretion of cytokines (e.g. TGF‐β) and proteases (e.g. MMPs). The altered composition and mechanics of the ECM amplify mechanical signals to cancer cells, thereby regulating downstream factors such as hypoxia‐inducible factor 1α (HIF1α). These changes promote tumor progression by enhancing cell proliferation, invasion, survival, and treatment resistance. Figure 1 was created by biorender.com.

In cancer, the remodeling of ECM constitutes a dynamic process driven collectively by tumor cells, CAFs, and immune cells (Figure 1). CAFs contribute to ECM stiffening and fibrosis through the secretion of collagen, fibronectin (FN), and HA, coupled with LOX family enzyme‐mediated collagen crosslinking [21]. Meanwhile, tumor cells facilitate invasion via EMT and MMP‐dependent basement membrane degradation. Immune cells further remodel the ECM by releasing proteases (e.g., MMP9, elastase) and neutrophil extracellular traps (NETs) [13]. Such remodeling activates prometastatic signaling pathways while establishing physical barriers that impede drug penetration and immune cell infiltration, ultimately promoting tumor progression, metastasis, and therapy resistance [22].

Alterations in ECM architecture and stiffness represent one of the hallmarks of cancer. Excessive deposition of collagen subtypes (e.g., COL1A1, COL4, COL6) serves as the primary driver of ECM stiffening, while LOX‐mediated collagen crosslinking directly increases matrix rigidity by enhancing the mechanical strength of fibers [23, 24]. The stiffened ECM establishes physical barriers that impede the infiltration and motility of immune cells, including chimeric antigen receptor T (CAR‐T) cells, resulting in reduced quantities of infiltrating CD8+ T cells within solid tumors [25, 26]. Furthermore, elevated ECM stiffness absorbs water and compresses vasculature, leading to increased interstitial fluid pressure (IFP) that obstructs intratumoral penetration of chemotherapeutic agents or immunotherapeutic drugs [27, 28, 29]. Studies have demonstrated that degradation of collagen or HA can improve drug distribution [30, 31, 32]. Furthermore, the increased stiffness of the ECM compresses blood vessels, leading to hypoxia and activation of the HIF‐1α signaling pathway, which promotes tumor cell adaptation and invasiveness [33]. Hypoxia upregulates the level of enzymes such as LOX through HIF‐1α, promoting collagen cross‐linking and ECM stiffening, thereby establishing a positive feedback loop [34, 35]. Additionally, hypoxia enhances the activity of MMPs, accelerating ECM degradation and releasing proangiogenic factors, which subsequently reduce immune cell infiltration and recruit immunosuppressive cells [36, 37, 38].

In addition to its mechanical barrier function, ECM can influence tumor progression through mechanotransduction signaling pathways. In gastric cancer (GC), increased ECM stiffness activates the RhoA/ROCK1 pathway, promoting MSCs to transfer mitochondria to tumor cells via microvesicles, thereby suppressing mitophagy and enhancing chemoresistance [39]. Studies indicate that ECM stiffening activates focal adhesion kinase (FAK) through integrin engagement, subsequently regulating downstream pathways such as PI3K/Akt and STAT3, which promote tumor invasion and stemness [40, 41]. For instance, sulfated ECM enhances EMT and stemness in lung cancer cells via the integrin β1/EGFR/PI3K axis [34]. Collagen, through discoidin domain receptor 1 (DDR1), activates STAT3, augmenting the invasiveness and metastasis of prostate cancer [42]. Furthermore, mechanical stretching of the ECM triggers Piezo1 calcium channels, inducing calcium signaling that upregulates the transcription factor Osr2 via cAMP response element‐binding protein, leading to CD8+ T cell exhaustion [43]. Research demonstrates that yes‐associated protein (YAP)/transcriptional coactivator with PDZ‐binding motif (TAZ) activation upregulates cyclins, antiapoptotic proteins, and stemness markers (e.g., SOX2, OCT4), thereby reinforcing chemoresistance [44, 45]. Notably, while YAP is widely recognized for mediating immune evasion via PD‐L1 upregulation, a recent study revealed that under specific mechanical conditions (e.g., 14 kPa stiffness or Piezo1 activation), YAP concurrently upregulates PD‐L1 and C–X–C motif chemokine ligand 10 (CXCL10), the latter recruiting CD8+ T cells and fostering an “immune‐responsive microenvironment” [46]. In animal models, Piezo1 agonists combined with anti‐PD‐1 therapy significantly increased CD8+ T cell infiltration and reduced metastatic liver tumor volume [46]. Thus, further investigation is warranted to elucidate the precise regulatory mechanisms of YAP nuclear translocation downstream of Piezo1 activation, particularly how YAP's dual role (protumorigenic vs. tumor‐suppressive) is modulated by mechanical cues across different cancer types.

The diverse components of ECM participate in tumor initiation and progression through complex heterogeneous functions, which are primarily manifested in the following aspects. As the most abundant ECM protein, collagen exhibits distinct functions depending on its subtypes and structural organization. For instance, Type I collagen (Col I) promotes tumor stiffness and activates the integrin/FAK signaling pathway, thereby enhancing cancer cell survival and invasive capabilities [25, 47]. COL VI upregulates HIF‐1α to participate in tumor angiogenesis, facilitating metastasis in lung cancer, glioblastoma (GBM), and breast cancer [48, 49, 50]. In contrast, Type III collagen (Col III) maintains tumor dormancy through the DDR1/STAT1 signaling pathway, suppressing metastatic growth [47]. Intact collagen IV (iCol IV) inhibits the DDR1/NF‐κB pathway, slowing pancreatic tumor progression [51]. As a key component of the basement membrane, laminin demonstrates opposing roles at different stages of tumor development. In early‐stage tumors, laminin preserves epithelial integrity and inhibits EMT, while regulating cell attachment through integrin and nonintegrin receptors [52, 53]. However, in advanced tumors, LAMC2 drives tumor metastasis by modulating the TGF‐β signaling pathway, and its defective anchoring function promotes progression in breast and brain cancers [54, 55]. In most human cancers, alterations in the expression of specific LM chains are confirmed to be associated with poor prognosis [56]. FN exhibits location‐specific functions in tumors. Within the tumor, FN modulates tumor immunity by activating tumor‐infiltrating lymphocytes and promotes macrophage migration via the SFK–FAK/colony‐stimulating factor 1 (CSF‐1)R signaling pathway [57, 58]. Conversely, in peritumoral tissues, FN enhances invasion through α5β1 integrin‐mediated FAK/Src activation [59]. Studies have shown that in dormant breast cancer, FN maintains cell survival through αvβ3/α5β1 integrin adhesion [60, 61]. The function of HA is highly dependent on its molecular weight, displaying significant heterogeneity with changes in its polymeric state. High‐molecular‐weight HA (HMW HA) maintains ECM homeostasis and inhibits tumor metastasis [62, 63]. Notably, in mouse models, HMW HA accumulation is accompanied by COL deposition and increased hypoxia, promoting tumor drug resistance [64]. In contrast, low‐molecular‐weight HA induces Toll‐like receptor (TLR)/CD44‐mediated proinflammatory signaling, facilitating immune evasion, and increases IFP, thereby limiting drug delivery [31, 65]. In summary, the heterogeneous functions of ECM components provide abundant therapeutic targets for tumor treatment but also present challenges, necessitating more in‐depth basic research and the development of more precise targeting strategies.

### 2.2 Cancer‐Associated Fibroblasts

CAFs represent the most abundant stromal cell population within the TME, originating from the activation and transformation of normal fibroblasts, pericytes, adipocytes, and other cell types through stimulation by tumor cells or inflammatory factors. CAFs exhibit remarkable heterogeneity and plasticity, actively participating in tumor progression through the secretion of cytokines, exosomes, and metabolic byproducts, as well as through ECM remodeling, thereby facilitating intricate interactions with tumor cells and immune cells [66]. CAFs demonstrate substantial functional and molecular heterogeneity, encompassing both tumor‐promoting subtypes (e.g., myofibroblastic CAFs [myCAFs] and inflammatory CAFs [iCAFs]) and tumor‐restraining subtypes (e.g., antigen‐presenting CAFs [apCAFs]). These distinct subpopulations may exert opposing effects on therapeutic outcomes, presenting significant challenges for the development of CAF‐targeted cancer therapies [67, 68].

#### 2.2.1 T Cells

**FIGURE 2:**
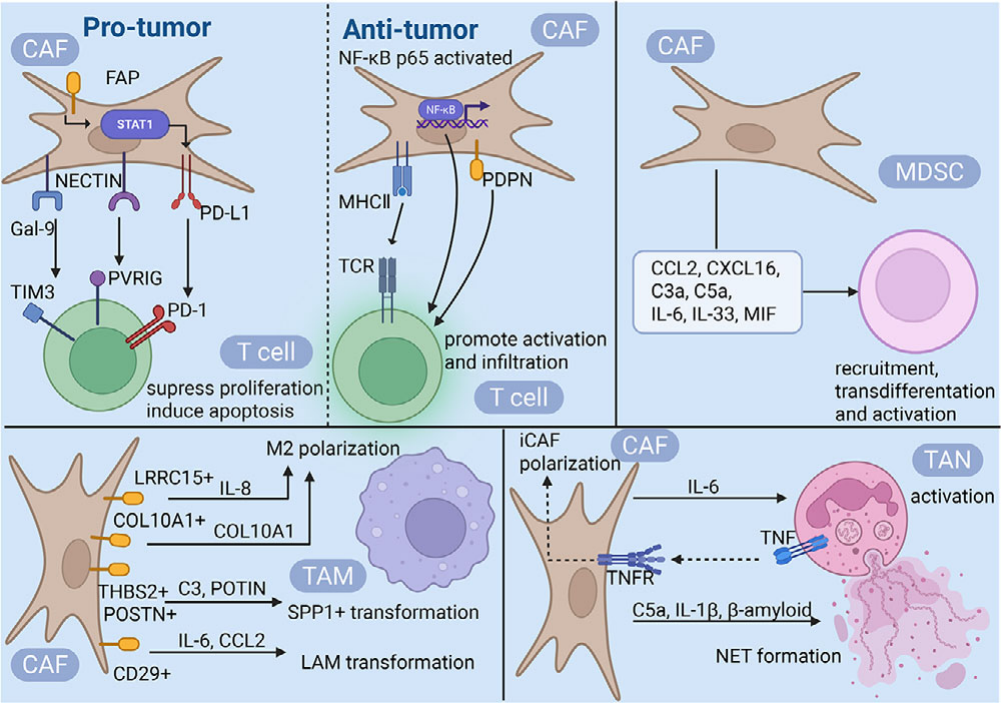
The interaction between CAFs and immune cells in the TME. On the one hand, CAFs directly suppress T cell function and induce apoptosis by expressing molecules such as FAP, Gal‐9, and PVRIG. On the other hand, CAFs with activated NF‐κB pathways and high expression of MHC‐II and PDPN promote T cell activation and TME infiltration. CAFs recruit and activate myeloid‐derived suppressor cells (MDSCs) by secreting factors like IL‐6 and CCL2, while also inducing macrophage polarization toward the M2 type (TAMs), thereby synergistically suppressing antitumor immunity. Additionally, CAFs influence the activation and function of neutrophils (TANs) through cytokine release. Figure 2 was created by biorender.com.

Numerous studies have demonstrated that CAFs can suppress effector T cell function through the production of immunosuppressive factors such as IL‐6 and CXCL12, as well as via PD‐1/PD‐L1 receptor signaling pathways [69, 70, 71, 72, 73] (Figure 2). In recent years, research has become increasingly refined, delving into distinct CAF subtypes and their spatial distribution across various cancer types. Fibroblast activation protein (FAP)+ CAFs have been shown to upregulate PD‐L1 expression by stabilizing STAT1 protein, thereby inhibiting CD8+ T cell cytotoxicity and promoting T cell exhaustion (e.g., through upregulation of inhibitory receptors like TIM3 and LAG3) [74]. Research indicates that FAP+ CAFs are enriched in the core of hepatocellular carcinoma (HCC) with a unique stromal architecture characterized by abundant intratumoral stroma but lacking a fibrotic rim, which correlates with poorer prognosis [75]. Targeting FAP+ CAFs (e.g., via FAP inhibition) combined with anti‐PD‐1 therapy demonstrates superior tumor regression compared with monotherapy in HCC models [76]. Extensive evidence suggests that FAP+ CAFs and inflammatory niches are prevalent across multiple cancer types, including non‐small cell lung cancer (NSCLC) [76, 77], colorectal cancer (CRC) [78, 79], head and neck squamous cell carcinoma (HNSCC) [80], and pancreatic ductal adenocarcinoma (PDAC) [8, 9], indicating broad clinical relevance. A notable finding reveals that dual targeting of mesothelin‐high PDAC cells and FAP‐expressing CAFs in the TME significantly enhances therapeutic efficacy against PDAC [81]. Recently, a novel CAF subset termed “T cell‐inhibiting CAFs” (TinCAFs) was identified. These TinCAFs preferentially localize near T cells in the TME and interact with T cell surface receptors through nectin cell adhesion molecule 2 (NECTIN2), markedly suppressing effector T cell proliferation and function while promoting T cell exhaustion. Notably, high NECTIN2 expression correlates with poorer survival in other malignancies (e.g., breast cancer, lung cancer, acute myeloid leukemia [AML]), warranting further investigation across cancer types [82]. In HNSCC, major histocompatibility complex Class I (MHC)–IhiGal‐9+ CAFs restrict CD8+ T cell infiltration into tumor regions and induce CD8+ T cell dysfunction through high expression of MHC‐I and Galectin‐9 (Gal‐9). Gal‐9 further suppresses CD8+ T cell antitumor activity via interaction with receptors such as TIM3 [83]. However, emerging evidence suggests certain CAF subsets may promote antitumor immunity. In GC, apCAFs expressing high levels of MHC Class II molecules were identified, showing preferential localization near tertiary lymphoid structures (TLSs). These apCAFs enhance T cell activation and drive macrophage polarization toward a proinflammatory phenotype, thereby amplifying antitumor immunity [84, 85, 86]. Baseline tumors from immunotherapy responders across multiple cancer types exhibit higher apCAF infiltration, suggesting apCAF levels may predict clinical benefit from immune checkpoint blockade therapy [84, 85, 86]. In prostate cancer, lymphocyte‐associated CAF (Lym‐CAF) enhances the infiltration of CD8+ T cells within tumors and upregulates the level of their activation markers (CD25, CD69) and effector molecules (granzyme B, interferon [IFN]‐γ), thereby inhibiting tumor growth [87]. Furthermore, podoplanin (PDPN)‐high CAF subtypes enriched in immunomodulatory pathways demonstrate significantly better prognosis [88]. PDPN‐positive CAFs are also associated with increased lymphocyte and macrophage infiltration in other cancer types [89, 90, 91].

#### 2.2.2 Macrophages

CAFs are activated during early tumorigenesis and express a series of NF‐κB‐dependent proinflammatory genes (e.g., CXCL1, CXCL2, IL‐6, IL‐1β, COX‐2, etc.) that serve as chemotactic signals to directly recruit immune cells such as macrophages into the TME [35, 92, 93, 94] (Figure 2). A recent study demonstrated that in GBM patients refractory to anti‐PD‐1 therapy, the formation of immunosuppressive macrophages depends on an leucine‐rich repeat‐containing protein 15 (LRRC15)‐high CAF subpopulation (myoCAFs). Genetic ablation of LRRC15 or treatment with anti‐LRRC15 antibodies reduced M2‐like macrophage infiltration while enhancing CD8+ T cell activation and tumoricidal function [94]. Furthermore, CAF‐secreted COL10A1 and sialylated glycans bind to monocyte surface receptors, inducing their differentiation into immunosuppressive TAMs (CD163+CD206+) [95, 96]. Thrombospondin 2 (THBS2)+ matrix CAFs (mCAFs) secrete complement component 3 (C3), while POSTN+ CAFs produce periostin (POSTN), collectively driving macrophage polarization toward immunosuppressive SPP1+ TAMs [97, 98]. Under hypoxic conditions, CAFs stabilize HIF2 level and upregulate macrophage migration‐related genes (e.g., Mmp9, Cd74) through paracrine signaling, facilitating macrophage recruitment into the TME and promoting M2 polarization [99]. Beyond secretory factors, CAFs upregulate VCAM‐1 level in CRC cells via IL‐6, thereby enhancing monocyte‐CRC cell adhesion [92]. In addition to conventional M2 subtypes, emerging research has revealed intricate associations between CAFs and other macrophage subsets in the TME. In triple‐negative breast cancer (TNBC), FAP+CD29+ iCAFs recruit monocytes via the CXCL12–CXCR4 axis and induce their differentiation into TREM2highSTAB1+ lipid‐associated macrophages (LAMs) through secretion of IL‐6 and CCL2. These LAMs potently suppress T cell activation and proliferation while upregulating inhibitory checkpoint molecules (e.g., PD‐1, CD25), thereby shaping an immunosuppressive TNBC microenvironment [100].

#### 2.2.3 Myeloid‐Derived Suppressor Cells

In certain cancers, CAFs attract monocytes by secreting chemokines such as CCL2 and CXCL16, promoting their migration into the TME and differentiation into myeloid‐derived suppressor cells (MDSCs) [101, 102]. A study on breast cancer reported that following doxorubicin (DOX) treatment, CAFs upregulate complement factors (e.g., C3a/C5a), recruiting MDSCs to metastatic sites via C3aR/C5aR1 to form an immunosuppressive microenvironment. Targeting complement signaling reduces MDSC infiltration. Additionally, CAFs secrete cytokines to activate metabolic pathways [103]. For instance, in intrahepatic cholangiocarcinoma, CAFs activate the 5‐lipoxygenase pathway in MDSCs by secreting IL‐6 and IL‐33, promoting leukotriene B4 production, thereby enhancing tumor stem cell properties and chemotherapy resistance [104]. In HCC models, CD36+ CAFs activate the lipid peroxidation/p38/CEBPs pathway via oxidized low‐density lipoprotein uptake [105], upregulating macrophage migration inhibitory factor expression to recruit CD33+ MDSCs and enhance their immunosuppressive functions [105].

#### 2.2.4 Tumor‐Associated Neutrophils

HCC‐CAFs recruit neutrophils via secretion of CXCL12, enhance their infiltration into tumors, and activate STAT3 signaling through IL6, thereby inducing PDL1 expression and fostering an immunosuppressive microenvironment in HCC. In PDAC, CAFs recruit neutrophils via the CXCL1–CXCR2 signaling axis and activate them through transmembrane TNF–TNFR2 interactions, establishing a feed‐forward loop that drives iCAF polarization and fosters an immunosuppressive TME [106]. In addition, CAFs drive NET formation by neutrophils through secretion of amyloid‐β [66]. NETs are web‐like structures composed of DNA fibers, histones, and antimicrobial proteins released by activated neutrophils, which significantly promote tumor growth and metastasis by enhancing angiogenesis, tumor cell migration and invasion, as well as trapping circulating tumor cells [107]. A recent study demonstrates that fibroblast growth factor 19 (FGF19)‐induced iCAFs promote CRC liver metastasis by activating neutrophils through the secretion of complement C5a and IL‐1β, thereby inducing NET formation [108].

### 2.3 Mesenchymal Stem Cells

MSCs are a class of multipotent nonhematopoietic stromal cells, first isolated and characterized by Friedenstein et al. in 1974 from murine bone marrow. Numerous studies have demonstrated that MSCs promote tumor progression through the secretion of growth factors (e.g., FGF, hepatocyte growth factor [HGF]) and extracellular vesicles (EVs), which activate prosurvival signaling pathways (e.g., PI3K/AKT) in tumor cells [109, 110, 111, 112, 113]. In pancreatic cancer, the activation of the IL‐6/STAT3 pathway can be reversed by tocilizumab (IL‐6 receptor blockade) or STAT3 inhibitors, significantly suppressing tumor growth [112]. MSCs have also been shown to stimulate angiogenesis (e.g., in liver and ovarian cancers) via the secretion of vascular endothelial growth factor (VEGF), basic bFGF, and HGF, thereby enhancing tumor cell proliferation [114, 115, 116, 117, 118].

In the TME, once MSCs home to the tumor site, they can induce EMT through direct physical contact with tumor cells [119], or via paracrine signaling [120]. This process primarily depends on TGF‐β, leading to the downregulation of epithelial markers (e.g., E‐cadherin) and the upregulation of mesenchymal markers (e.g., N‐cadherin, vimentin), thereby enhancing migration and invasion of tumor cells [121, 122]. For instance, MSC‐derived TGF‐β promotes EMT in breast cancer cells via Smad signaling [123]. Studies have also shown that MSCs secrete MMPs (e.g., MMP‐2/9) to degrade the ECM, facilitating tumor cell invasion [124]. Coculture of MSCs with ovarian cancer cells upregulates MMP‐9 expression [125].

Furthermore, MSCs modulate the tumor immune microenvironment. They suppress CD4+ and CD8+ T‐cell proliferation and function by secreting nitric oxide, prostaglandin E2, TGF‐β, and indoleamine 2,3‐dioxygenase (IDO), inducing cell cycle arrest at the G1 phase [126, 127, 128, 129]. Research indicates that MSCs promote immune evasion in CRC by inducing CD8+ T‐cell exhaustion via hypersialylation (Siglec receptor binding) [130]. MSCs also enhance Treg generation through TGF‐β and IL‐10 secretion, maintaining immune tolerance [111, 131]. Additionally, MSCs impair NK cell function by upregulating FBP1 to inhibit glycolysis or downregulating NKG2D ligands (e.g., MICA/B), reducing NK cell activation [132, 133, 134]. M2 macrophage polarization, driven by MSC‐secreted IL‐6, IL‐10, and CCL2, further enhances immunosuppression [135, 136]. MSCs also regulate the TME via EV‐mediated miRNA transfer. For example, Lou et al. demonstrated that adipose tissue‐derived MSC exosomes carrying miR‐122 enhance the sensitivity of HCC cells to sorafenib [137]. In breast cancer, exosomal miRNAs (e.g., miR‐222/223) promote quiescence by modulating cell cycle‐related proteins [138]. Bone marrow MSC‐derived exosomes upregulate S100A4, enhancing proliferation, invasion, and chemoresistance in AML cells [139].

Mesenchymal stromal cells (MSCs) exhibit a context‐dependent, dual role in tumor progression. While substantial evidence indicates that MSCs can be recruited to tumor sites and, upon interaction with the TME, be co‐opted into protumorigenic cancer‐associated MSCs that support growth, invasion, and metastasis, a parallel body of research highlights their inherent antitumor potential. Naive or engineered MSCs can directly inhibit tumor cell proliferation by secreting tumor‐suppressive factors (e.g., DKK‐1, TRAIL) or activating apoptotic pathways [115, 140]. Lu et al. demonstrated that human bone marrow MSCs suppress GBM U251 cell proliferation and induce apoptosis via caspase‐9/3 activation and Bcl‐2 downregulation [141]. Melatonin pretreatment enhances MSC survival in the TME and potentiates their anti‐GBM effects, partly due to EMT reversal [142]. Recent studies found that human umbilical cord MSC‐derived small EVs (hucMSC‐sEVs) effectively inhibit GC cell proliferation, migration, and invasion, with Pentraxin 3 identified as a key mediator [143]. Fusion of hucMSC‐sEVs with neutrophil membranes generates Neu/MSC‐sEVs [143], which exhibit enhanced tumor targeting, prolonged circulation, and improved therapeutic efficacy while evading mononuclear phagocyte system clearance [143]. Given the functional heterogeneity of MSCs from different tissue sources and the incomplete understanding of their pro‐ and antitumor mechanisms, further research is needed to elucidate their complex molecular regulation.

## 3 Tumor Stroma as a Therapeutic Target for Cancer

Building upon the foundational biology outlined in the previous section, this chapter methodically examines the therapeutic arsenal targeting each major stromal component. The paragraph begins with the dense ECM, reviewing strategies from direct enzymatic degradation (e.g., targeting HA, collagen) and inhibition of matrix‐modifying enzymes (e.g., LOX, MMPs) to interfere with critical downstream signaling pathways (e.g., TGF‐β, integrins, DDR1). We then shift focus to the aberrant tumor vasculature, analyzing the evolution of therapeutic concepts from antiangiogenesis and vascular disruption to the more nuanced approaches of vascular normalization and the induction of high endothelial venules (HEVs). Subsequent sections delve into the complex targeting of cellular stromal components: CAFs, covering depletion, activation inhibition, and reprogramming strategies; TAMs, exploring depletion, repolarization, and engineered cell therapies like CAR‐M; and the multifaceted targeting of ECs, MSCs, and pericytes. For each target, we integrate key findings from preclinical studies and highlight the progress and outcomes of relevant clinical trials, offering a translational perspective on the journey from bench to bedside.

### 3.1 Targeting ECM

#### 3.1.1 Direct ECM Component Targeting

The tumor stroma is rich in ECM components, such as HA, collagen, LOXs, and tenascin‐C (TNC), which can directly promote or activate downstream signaling pathways leading to tumor cell proliferation or immune suppression. Therefore, directly targeting ECM components and their associated signaling pathways represents a theoretically feasible strategy with significant clinical therapeutic potential (Table 1). Numerous animal studies have demonstrated that enzymatic degradation of these components, small‐molecule drug interventions, and nanomaterial‐based delivery systems can effectively degrade the ECM and suppress tumor progression, providing valuable insights for clinical treatment approaches [7, 22, 144].

**TABLE 1:** ECM‐targeted therapies for cancer.

Target	Drug name	Subject tumors	Combination	Phase	Status	NCT number
Hyaluronidase	PEGPH20	Stage IV pancreatic cancer	Gemcitabine	II	Completed	NCT01453153
		HA high metastatic PDAC	Pembrolizumab	II	Unknown	NCT03634332
		Advanced solid tumors	Alone	I	Completed	NCT00834704
		Chemotherapy‐resistant pancreatic cancer	Avelumab	Early I	Terminated	NCT03481920
	Daratumumab hyaluronidase‐fihj	MM	TTI‐622	I	Active	NCT05139225
	Hyaluronidase	Relapsed/​refractory CLL	Rituximab, venetoclax	I	Active	NCT03467867
		Stage III–IV melanoma	Rituximab	II	Active	NCT03719131
Angiotensin II receptor	Losartan	Localized pancreatic cancer	FOLFIRINOX + chemoradiotherapy	II	Active	NCT03563248
		Metastatic PDAC	FOLFIRINOX + 9‐Ing‐41 (glycogen synthase kinase‐3β inhibitor)	II	Recruiting	NCT05077800
		Osteosarcoma	Sunitinib	I	Recruiting	NCT03900793
		Localized pancreatic cancer	Nivolumab, FOLFIRINOX, and SBRT	II	Active	NCT03563248
		Pancreatic cancer	FOLFIRINOX and elraglusib	II	Active	NCT05077800
LOX	Simtuzumab	Metastatic pancreatic adenocarcinoma	Gemcitabine	II	Completed	NCT01472198
		CRC	FOLFIRI	II	Terminated	NCT01479465
		Myelofibrosis	Ruxolitinib	II	Completed	NCT01369498
	PXS‐5505	Myelofibrosis	Alone	I/II	Completed	NCT04676529
	Tetrathiomolybdate	CRC	Irinotecan, 5‐fluorouracil, leucovorin	II	Completed	NCT00176774
MMPs	Prinomastat	Glioblastoma multiforme	Temozolomide	II	Completed	NCT00004200
		Metastatic or recurrent NSCLC	Cisplatin, gemcitabine hydrochloride	III	Completed	NCT00004199
		Metastatic PC	Endocrine‐modulating drug therapy, mitoxantrone hydrochloride, prednisone	III	Completed	NCT00003343
	Andecaliximab	Advanced solid tumors	Alone and in combination with chemotherapy	I	Completed	NCT01803282
		Gastric or gastroesophageal junction adenocarcinoma	mFOLFOX6	I	Colmpleted	NCT02545504
	BT1718	Advanced solid tumors	Alone	I/II	Completed	NCT03486730
Tenascin‐C	131Iodine‐tenatumomab	Tenascin‐C positive cancer	±Dostarlimab	I	Terminated	NCT02602067
	I‐81C6	GBM	Neuradiab and radiotherapy	III	Completed	NCT00615186
	Tenarad	Solid tumor and lymphomas	Alone	I/II	Completed	NCT01240720
	F16‐IL2	NSCLC	Nivolumab, fixed dose	I/II	Unknown	NCT05468294
Integrin blocking (type)	Abituzumab (av)	PC	Alone	I	Completed	NCT00958477
		K‐ras wild yype metastatic CRC	Cetuximab, irinotecan	I/II	Completed	NCT01008475
		Liver metastases	Alone	I	Completed	NCT00848510
		Asymptomatic or mildly symptomatic metastatic castrate‐resistant PC	Alone	II	Completed	NCT01360840
	Volociximab (αvβ1)	Metastatic pancreatic cancer	Gemcitabine	II	Completed	NCT00401570
		NSCLC	Carboplatin, paclitaxel	I	Completed	NCT00654758
		NSCLC	Carboplatin, paclitaxel, bevacizumab	I	Completed	NCT00666692
		Metastatic melanoma	Dacarbazine	II	Completed	NCT00099970
	Etaracizumab (αvβ3)	Advanced CRC	Alone	I/II	Completed	NCT00027729
		Advanced solid tumors or lymphoma	Alone	I	Completed	NCT00049712
		Unresectable or metastatic KC	Alone	I/II	Completed	NCT00684996
Integrin targeting (type)	CEND‐1 (αvβ3, αvβ5)	Advanced metastatic pancreatic ductal adenocarcinoma	Alone	I/II	Completed	NCT05052567
		Metastatic pancreatic cancer	Nab‐paclitaxel, gemcitabine	I	Completed	NCT03517176
		Pancreatic, colon cancer and appendiceal cancers	Panitumumab, folfirinox	I/II	Active	NCT05121038
	ProAgio (αvβ3)	Advanced pancreatic cancer	Alone	I	Active	NCT05085548
		Metastatic TNBC	Gemcitabine	I/II	Recruiting	NCT06460298
		Advanced/metastatic CRC	FOLFIRI, bevacizumab	I	Recruiting	NCT06867822
	7HP349 (α4β1, αLβ2)	Locally advanced or metastatic cancers	Ipilimuma, nivolumab	I/II	Recruiting	NCT06362369
TGF‐β	Galunisertib	Solid tumors	Alone	I	Completed	NCT02304419
		Metastatic pancreatic cancer	Durvalumab	I	Completed	NCT02734160
		Advanced or metastatic unresectable pancreatic cancer	Gemcitabin, placebo	I/II	Completed	NCT01373164
	SHR1701	Advanced solid tumors and B‐cell lymphomas	SHR2554	I/II	Recruiting	NCT04407741
		Advanced solid tumors	Alone	I	Completed	NCT04324814
		Pancreatic cancer	Alone	I/II	Completed	NCT04624217
DDR1		Advanced malignancies	Alone	I	Recruiting	NCT05753722
FAK	GSK225609	Advanced solid tumors	Trametinib	I	Completed	NCT01938443
		Advanced pancreatic cancer	Trametinib	II	Completed	NCT02428270
		Progressive meningiomas	Vismodegib, capivasertib, abemaciclib	II	Recruiting	NCT02523014
	CT‐707	Advanced pancreatic cancer	Toripalimab, gemcitabine	I/II	Recruiting	NCT05580445
		Advanced solid tumors with KRAS p.G12C mutation	SY‐5933	I/II	Recruiting	NCT06970132
	Defactinib	Nonhematologic Malignancies	Alone	I	Completed	NCT01943292
		Advanced cancer	Pembrolizumab, gemcitabine	I	Completed	NCT02546531
		Thyroid cancer	Avutometinib	II	Recruiting	NCT06007924
		Advanced ovarian cancer	Paclitaxel	I	Completed	NCT01778803
Wnt/β‐catenin	Vitamin D3	Pancreatic cancer	Dostarlimab, mFOLFIRINOX treatment, regimen LV5FU2	II	Recruiting	NCT06757244

Abbreviations: PDAC: pancreatic ductal adenocarcinoma, MM: multiple myeloma, CLL: chronic lymphocytic leukemia, GAMMA‐1: intravenous injection, GBM: glioblastoma multiforme, HNSCC: head and neck squamous cell carcinoma, NSCLC: non‐small‐cell lung carcinoma, CRC: colorectal cancer, KC: kidney cancer, PC: prostate cancer.

*Data sources*—ClinicalTrials.gov.

##### 3.1.1.1 Hyaluronan

HA is a key component of the ECM. Over two decades ago, research teams began exploring intratumoral injections of hyaluronidase to reduce tumor IFP [145]. Since then, the use of hyaluronidase to remodel the tumor ECM has attracted significant research interest. In an osteosarcoma xenograft model, intratumoral injection of hyaluronidase was experimentally confirmed to reduce IFP by 50% and increase the intratumoral concentration of liposomal DOX to fourfold that of the control group [31]. Although direct intratumoral hyaluronidase injection showed some efficacy, its clinical application faced challenges. Subsequently, researchers developed PEGylated recombinant human hyaluronidase (PEGPH20). In solid tumor animal models, PEGPH20 not only degraded HA but also remodeled intratumoral vessels [146]. Following this, antitumor studies based on PEGPH20 have proliferated. The fundamental strategy remains the direct degradation of HA in animal models to improve drug distribution and enhance the efficacy of both chemotherapy and immunotherapy [32, 147, 148]. Multiple clinical trials investigating PEGPH20 as an adjunctive agent have demonstrated that targeting tumors with high HA content in the ECM can sensitize them to existing therapies and extend patient survival [149, 150]. However, a Phase III trial with an expanded sample size revealed that PEGPH20 provided only limited survival benefit across the broader patient population of metastatic pancreatic cancer [151, 152]. This outcome starkly contrasts with the underlying biological rationale and earlier Phase II data suggesting enhanced efficacy in tumors with high HA content [149, 150]. Critically, the failure to improve survival in an unselected population underscores the absolute necessity of biomarker‐driven patient selection. The prespecified hypothesis, supported by preclinical models, is that the benefit of HA degradation is contingent on the presence of a HA‐rich stroma (NCT03634332). This strongly suggests that using validated immunohistochemical or quantitative image analysis methods to assess tumor HA burden is a clinically feasible and necessary strategy for patient selection. In one study, the addition of PEGPH20 to a combination of FAK inhibitors and anti‐PD‐1 (aPD‐1) therapy reduced the recruitment of immunosuppressive cells, such as CXCR4‐expressing myeloid cells [153]. Beyond PEG‐based formulations, hyaluronidase encapsulated in iron oxide nanoparticles (NPs) has also been shown to degrade tumor HA and reduce tumor burden in mice, though this approach remains far from clinical application [153].

##### 3.1.1.2 Collagen

In addition to HA, collagen is another major constituent of the ECM. Current research focuses on inhibiting collagen production and preventing its cross‐linking with other components in the TME [154]. Similar to studies on HA, Eikje et al. attempted direct collagen degradation via intratumoral injection of collagenase, reporting analogous outcomes—namely, a reduction in tumor IFP [155]. However, this straightforward enzymatic approach does not necessarily translate into clinical benefits. Loss of structural collagen may even facilitate tumor metastasis, and certain types of collagenases have been associated with tumor progression [156, 157]. Given the uncertainty surrounding direct collagen degradation as an anticancer strategy, an alternative approach involves intervening in collagen biosynthesis. Losartan (LOS), a well‐established angiotensin II receptor antagonist, has been found to exert noncanonical pharmacological effects. Diop‐Frimpong et al. demonstrated its ability to inhibit Col I production by CAFs, an effect that exhibits a dose‐dependent trend across multiple animal tumor models [158]. Through this mechanism, LOS enhances the distribution and therapeutic efficacy of intratumorally administered agents [159, 160]. Subsequent research has focused on optimizing LOS delivery to improve tumor penetration, utilizing various carrier systems such as shrinkable NPs, paclitaxel‐loaded pH‐sensitive liposomes, peptide hydrogels, among others [161, 162]. Beyond its ECM‐targeting actions, LOS also exhibits intrinsic antitumor effects, including but not limited to blocking the recruitment of CCR2‐positive monocytes and suppressing IGF‐1 signaling [163, 164]. With the advent of targeted therapy and immunotherapy, the specific role of LOS in ECM degradation has been further elucidated. Multiple studies have demonstrated that in animal models, LOS inhibits collagen deposition in nonalcoholic steatohepatitis‐driven HCC, alleviates hypoxia in TNBC, promotes the infiltration of CD8+ T cells in anti‐PD‐1 therapy, and suppresses metastasis‐associated fibroblasts while reducing tumor stiffness [158, 165, 166]. Owing to its effective collagen‐targeting and well‐established safety profile, several clinical trials have been conducted to evaluate the clinical benefits of LOS. Encouragingly, the addition of LOS to the FOLFIRINOX chemotherapy and radiotherapy regimen was found to suppress the expression of immunosuppression‐related genes and reduce Tregs in tumors [167]. A single‐arm study further showed that LOS combined with chemoradiotherapy could facilitate downstaging in locally advanced PDAC [168]. However, a recently published Phase II clinical trial reported that LOS combined with chemotherapy did not improve overall or median survival in an unselected population of PDAC patients [169]. This result highlights the critical gap between preclinical promise and clinical reality: the benefit of reducing collagen synthesis may only manifest in patients with significant pre‐existing collagen deposition. Consequently, the search for predictive biomarkers must be hypothesis driven. For LOS, the most logical candidate is the baseline level of tumor collagen. Future investigations should therefore prioritize the validation of stromal collagen content as a predictive biomarker through retrospective analysis of tumor samples from completed trials and prospective integration of biomarker‐driven stratification (e.g., based on histological assessment of fibrosis) in ongoing trials (such as NCT03900793, NCT03563248, NCT05077800).

Among therapeutic agents targeting collagen in the ECM, LOS is not the only option. Pirfenidone (PFD), a clinically approved antifibrotic drug primarily used for idiopathic pulmonary fibrosis (IPF) and other fibrotic diseases, has attracted interest for its potential antitumor effects due to its antifibrotic properties [170, 171, 172]. The drug acts by inhibiting the production of multiple cytokines involved in collagen signaling—such as TGF‐β1, platelet‐derived growth factor (PDGF), IL‐1β, and TNF‐α—which are also critically implicated in tumor ECM formation [173]. Consequently, PFD exhibits direct antitumor activity. Numerous animal studies have confirmed its ability to modulate tumor stiffness, alleviate immunosuppression, inhibit tumor cell proliferation, and suppress EMT, indicating its promising potential in oncology [174, 175, 176, 177, 178]. In clinical practice, PFD was initially applied as a perioperative protective agent during tumor surgery to reduce postoperative and chemoradiotherapy‐related acute exacerbations of IPF, as well as radiation‐induced lung injury [179, 180, 181]. However, its direct therapeutic benefit in cancer treatment remains unclear. A clinical trial evaluating PFD combined with first‐line chemotherapy for advanced NSCLC (NCT03177291) has been completed, though no results have been disclosed. Other trials investigating PFD as a radiosensitizer in HNSCC or in combination with atezolizumab for NSCLC (NCT06142318, NCT04467723) are still recruiting, while two additional trials combining PFD with immunotherapy, VEGFR inhibitors, and chemotherapy have not yet begun patient recruitment (NCT06484153, NCT07161791). Pamrevlumab, which has a mechanism similar to PFD and is also indicated for IPF, failed to improve patient survival in two Phase II studies when combined with gemcitabine plus nab‐paclitaxel or FOLFIRINOX (NCT03941093, NCT04229004) [182]. These outcomes underscore the urgent need for novel therapeutic agents and improved drug delivery strategies. Through screening of traditional Chinese medicine, Li et al. identified halofuginone as an agent capable of directly inhibiting Col I deposition and promoting CD8+ T cell infiltration in ovarian tumors [28]. In a preclinical model of pancreatic cancer, engineered EVs loaded with PFD and miRNA were shown to suppress CAF activity and reduce tumor ECM content. Furthermore, a research team has developed dual‐targeting NPs capable of simultaneously blocking TGF‐β signaling and Col I production within tumors, thereby enhancing the penetration of DOX [183].

Although preclinical studies established a clear rationale for enzymatic or pharmacologic depletion of ECM components as a means to reduce interstitial pressure and improve drug delivery, clinical outcomes for both HA‐ and collagen‐targeted approaches have been inconsistent or disappointing. Several convergent explanations reconcile these observations. Mechanistically, both HA and collagen are biologically active, context‐dependent components. HA engages receptors such as CD44 to regulate endothelial and cancer‐cell behavior, and enzymatic cleavage generates low‐molecular‐weight HA fragments that can act as bioactive ligands altering inflammation and vascular permeability [147]. Likewise, collagen degradation yields bioactive fragments and disrupts the mechanical homeostasis of the tissue, with potential to activate integrin‐dependent promigratory and EMT programs [157]. These dual roles mean that acute matrix removal may transiently improve perfusion yet simultaneously release protumorigenic signals or remove structural restraints that limit invasion.

Beyond intrinsic biology, practical translational gaps are decisive. Many successful animal protocols use intensive or locally delivered enzyme exposure and tightly controlled sequencing, which creates a sustained window of enhanced penetration. Clinical regimens, by contrast, are constrained by systemic toxicity, immunogenicity, and logistical timing, and therefore may fail to align the period of maximal matrix depletion with the pharmacokinetic peak of cotherapies [146]. Furthermore, stromal compartments can mount rapid compensatory responses. For example, activated stromal cells can reconstitute HA or upregulate alternative matrix/cross‐linking pathways, so single‐agent ECM depletion is frequently transient unless the cellular sources or redundant pathways are cotargeted (e.g., PEGPH20 + gemcitabine strategies in preclinical models) [32, 151].

Finally, model and patient heterogeneity amplify failure risk. Preclinical models often have simpler, faster‐evolving stroma and lack the full immune and evolutionary complexity of human tumors [148]. Human lesions show wide variation in ECM abundance, spatial distribution, and cellular context [184]. Clinical trials typically recruit advanced, heavily pretreated patients whose resistance networks extend beyond delivery barriers [165]. And this variability directly underpins the mixed clinical outcomes observed with stroma‐targeting agents. For instance, the efficacy of HA‐depleting agents like PEGPH20 is strongly contingent on pre‐existing tumor HA abundance [149]. Clinical evidence is most robust for HA‐high tumors, defined by affinity histochemistry assays showing HA staining in ≥50% of the tumor surface area [150]. This biomarker was prospectively validated in the HALO 202 trial, where PEGPH20 combined with chemotherapy significantly improved progression‐free survival specifically in the HA‐high subgroup, but not in the overall intention‐to‐treat population [149]. Beyond bulk quantification of ECM components (e.g. total HA or collagen abundance), the spatial architecture of the tumor stroma also plays a critical role. In HCC, stromal archetypes defined by pathological image analysis and spatial proteomics (e.g., intratumoral FAP+ CAF‐rich vs. marginal C7+ CAF‐rich stroma) correlate with distinct biological functions and patient prognosis, suggesting that spatial mapping of stromal features could further refine patient selection. Therefore, future clinical translation of stromal therapies must prioritize biomarker‐stratified trial designs [75]. Realistic clinical assays include standardized immunohistochemistry for HA and collagen, digital pathology analysis of tumor stroma ratio, and emerging multiplex imaging or spatial transcriptomics to define prognostically relevant stromal architectures. Moving from unselected patient populations to biomarker‐defined cohorts is critical for demonstrating clear clinical benefit and advancing precision stromal therapy.

##### 3.1.1.3 LOXs, MMPs, TNC

As previously discussed, unlike HA and collagen that directly constitute the ECM, LOXs promote ECM deposition by cross‐linking collagen via integrin signaling [185]. Current research has identified various small‐molecule compounds and therapeutic agents capable of inhibiting LOX family activity, effectively blocking collagen cross‐linking in the tumor ECM both in vitro and in vivo. Examples include small‐molecule inhibitors (e.g. β‐aminopropionitrile [BAPN], PXS‐5505, LXG6403), LOX inhibitory antibodies, pterostilbene plus curcumin analogues, lipid nanovesicles, aminomethylenethiophenes, and methenamine hydrochloride [186, 187, 188, 189, 190, 191, 192, 193, 194, 195]. Similarly, suppressing upstream signals of LOX expression—such as macrophage‐derived oncostatin M and hepatitis transactivator protein X—can also restrict LOX levels, thereby achieving anti‐ECM deposition effects [196, 197]. Most LOX inhibitors remain in the preclinical stage. Among those that have entered clinical trials, simtuzumab, a monoclonal antibody originally developed for benign fibrotic diseases, is a notable example. However, its efficacy in suppressing tumor ECM deposition remains limited. Simtuzumab combined with gemcitabine failed to prolong survival in PDAC patients, and its addition to FOLFIRI chemotherapy did not benefit those with KRAS‐mutant colorectal adenocarcinoma [198, 199]. Tetrathiomolybdate, a nontargeted nanomaterial that inhibits LOX activity in a copper‐dependent manner, has completed a Phase II trial in combination with chemotherapy for colon cancer, though results have not been published (NCT00176774).

The MMP family also serves as a key regulator of ECM cross‐linking, playing a broad and dynamic role in ECM remodeling. Like LOXs, numerous specific and broad‐spectrum MMP inhibitors have been developed [200]. Batimastat was among the first broad‐spectrum MMP inhibitors to enter clinical trials. Although effective in mouse models, it showed no significant survival benefit in patients [201, 202]. Another broad‐spectrum inhibitor, marimastat, faced similar challenges, failing to provide benefit in multiple solid tumors while exhibiting notable musculoskeletal toxicity [203, 204, 205]. Other MMP inhibitors, including CGS‐27023A, tanomastat, prinomastat, and rebimastat, also demonstrated efficacy in animal studies but could not advance to clinical use due to toxicity and adverse effects [200]. Therefore, subsequent research targeting MMPs and the ECM has shifted toward extracting natural MMP inhibitors, developing monoclonal antibodies, and designing safer and more effective small‐molecule inhibitors—though these agents remain far from clinical application [206, 207, 208]. One small‐molecule inhibitor, BT1718, which recognizes and inhibits MT1‐MMP, has completed a Phase I/IIa trial in solid tumors and shown preliminary therapeutic effects, though larger patient cohorts are needed for further validation (NCT03486730).

TNC is a glycoprotein widely present in tumors. Its expression is upregulated during ECM stiffening and EMT, and it interacts with collagen and MMPs to promote ECM formation and foster an immunosuppressive microenvironment [209, 210]. Therefore, targeting TNC holds considerable clinical potential. Various strategies have been explored, including monoclonal antibodies, antibody–drug conjugates (ADC) and derivatives, NPs, short peptides, nucleic acid aptamers, and small RNAs [211]. These agents have shown efficacy in suppressing the growth of solid and hematologic tumors in vitro or in animal models. Several TNC‐targeting therapies have entered clinical trials, such as tenatumomab (NCT02602067), 131I‐81C6 (NCT00615186), Tenarad (NCT01240720), and F16‐IL2 (NCT05468294). However, these have only completed Phase I or II studies, and their therapeutic benefits require further evaluation. Beyond pharmacological targeting, one research group developed cytokine‐induced killer cells expressing a TNC‐specific CAR, which exhibited significant antitumor activity in mice [212].

##### 3.1.1.4 Integrins, Piezo1, TRPV4

Integrins also play a critical role in ECM formation, serving not only as structural connectors to ECM components but also as mediators of mechanical stress transmission. Cilengitide, an integrin antagonist targeting αvβ3 and αvβ5 integrins, disrupts integrin–ECM interactions and exhibits anti‐ECM effects across multiple solid tumor models [213]. However, a large Phase III clinical trial demonstrated that cilengitide failed to improve outcomes in glioma patients [214]. Its efficacy in other solid tumor types remains under investigation (NCT01118676). Similarly, volociximab—a monoclonal antibody against integrin α5β1—has encountered challenges comparable to those of cilengitide. Results from several clinical trials have yet to be disclosed (NCT00401570, NCT00516841). Beyond integrins, other mechanosensitive components such as Piezo1 and TRPV4 also contribute to ECM mechanotransduction. Studies have reported that their respective inhibitors, Dooku1 and GSK1016790A, show potential in suppressing ECM formation [215, 216]. These collective findings underscore the heterogeneity and the complexity of the ECM network, highlighting the need for more precise therapeutic targeting strategies.

#### 3.1.2 ECM Signaling

Beyond directly targeting existing ECM components, inhibiting ECM deposition or downstream signaling transduction represents another crucial dimension of ECM‐directed anticancer therapy (Table 1). Substantial evidence has established TGF‐β as one of the most pivotal drivers of ECM formation, and therapeutic strategies targeting TGF‐β have shown promising progress [217]. Currently, numerous clinical trials involving TGF‐β antibodies or its inhibitors are underway. For instance, galunisertib improved the total response rate to 32% in patients with locally advanced rectal cancer [218]. Another bifunctional TGF‐β inhibitor, SHR‐1701, has also demonstrated encouraging efficacy in patients with unresectable solid tumors [219, 220].

The DDR family, particularly DDR1, is widely expressed on various cell surfaces. In coordination with CD44, it enhances tumor cell stemness and suppresses immune infiltration upon collagen fiber stimulation [25, 221, 222]. Several preclinical studies have shown that NP‐delivered DDR1‐siRNA, the DDR1 inhibitor 7rh benzamide, the synthetic monoclonal antibody PRTH‐101, and DDR1 degradation via PROTAC technology can effectively block collagen‐induced DDR1 activation, laying the groundwork for clinical translation [223, 224, 225, 226].

In collagen signaling, HSP47 serves as another critical molecular chaperone during collagen deposition, targeting HSP47 with microRNA‐29a directly inhibits ECM accumulation [227, 228]. FAK, activated downstream of integrins, is regarded as another key regulator of the ECM. The FAK inhibitor GSK2256098 has shown efficacy in patients with *NF2*‐loss tumors [229], whereas another inhibitor, defactinib, exhibited limited activity in *NF2*‐loss solid tumors, underscoring the need for deeper mechanistic investigation into FAK inhibition [230, 231].

Vitamin D also participates in ECM formation. Studies indicate that vitamin D3 suppresses the Wnt/β‑catenin and mTOR signaling pathways in human uterine fibroid cells, thereby reducing ECM production [232]. Malignant tumor cells undergo apoptosis and exhibit decreased MMP expression upon vitamin D induction [233, 234]. In pancreatic cancer models, modified vitamin D analogs inhibit ECM formation [235]. Consequently, vitamin D is also being evaluated as an anti‐ECM agent in ongoing anticancer clinical trials (NCT06757244, NCT00238199, NCT03883919).

Additionally, the RORγ inhibitor GSK805 blocks the expression of ECM‐related genes and suppresses FN‐1 and collagen deposition in HCC stroma [236]. Minini et al. developed gold‐labeled iron oxide nanoflowers for photothermal therapy, which reduced ECM content in a preclinical cholangiocarcinoma model and enhanced cytotoxic T‐cell infiltration when combined with anti–PD‐1 therapy [237].

In summary, ECM represents one of the most dynamic and complex components of the tumor stroma. Throughout tumor initiation, progression, and posttreatment phases, the composition and structure of the ECM undergo continuous remodeling, posing significant challenges for ECM‐targeted therapeutic strategies. Existing research suggests that monotherapies targeting individual ECM components have shown limited success in oncology. As a highly dynamic and networked functional system, the ECM demands multidimensional and adaptive interventional approaches. In light of current therapeutic bottlenecks, future research should focus on enhancing the efficacy of existing agents, designing more efficient and rational delivery systems, and exploring synergistic drug combinations. Concurrently, the continued discovery of novel ECM‐related targets will deepen our understanding of its role in the TME, providing a solid theoretical and experimental foundation for translating basic research into clinical benefits.

### 3.2 Targeting Tumor Vasculature

Tumor vasculature within the tumor stroma plays a critical role in tumor initiation and progression. On one hand, tumor cells utilize the vascular network to obtain essential nutrients for proliferation and secrete bioactive factors to induce structural and functional remodeling of blood vessels. On the other hand, tumor cells exploit the vasculature as a conduit for metastasis, facilitating their dissemination to distant organs. As our understanding of the mechanisms underlying tumor vascular function continues to deepen, therapeutic strategies targeting tumor vasculature are also evolving, yet they still face significant challenges (Table 2).

**TABLE 2:** Major clinical trials targeting tumor vasculature.

Target	Drug name	Subject tumors	Combination	Phase	Status	NCT number
VEGF/VEGFR	Bemarituzumab	Solid tumors	Alone	IV	Completed	NCT01588184
		GC or CEJ adenocarcinoma	Chemotherapy	III	Active	NCT05052801
		Lung cancer	Erlotinib	II	Completed	NCT01562028
		Osimertinib lung cancer	Osimertinib	III	Recruiting	NCT04181060
		Lung cancer	Osimertinib	I/II	Completed	NCT02803203
		Ovarian cancer	Niraparib	I/II	Completed	NCT02354131
		HCC	Erlotinib	II	Completed	NCT01180959
	Olaparib	Hemotherapies ovarian cancer	Hemotherapies	III	Active, not recruiting	NCT02477644
		Enzalutamide, abiraterone acetate prostate cancer	Enzalutamide, abiraterone acetate	III	Active, not recruiting	NCT02987543
	Ramucirumab	Gastric adenocarcinoma	Paclitaxel	III	Completed	NCT01170663
		GC and adenocarcinoma	Alone	III	Completed	NCT00917384
		HCC	Alone	III	Completed	NCT01140347
		HCC	Alone	III	Completed	NCT02435433
		HCC	Alone	III	Completed	NCT02435433
	Sorafenib	HCC	Alone	III	Completed	NCT00692770
		RCC	Alone	III	Completed	NCT00586105
	Sunitinib	RCC	AGS‐003	II	Completed	NCT00678119
		RCC	Nivolumab, pazopanib, ipilimumab	I	Completed	NCT01472081
PDGF	Avapritinib	GIST	Alone	IV	Completed	NCT04825574
		GIST	Tyrosine kinase inhibitors	III	Completed	NCT05381753
		Locally advanced unresectable or metastatic GIST	Regorafenib	III	Completed	NCT03465722
	Telatinib	GC or HCC	Keytruda	II	Completed	NCT04798781
		Advanced GC	Cisplatin, Capecitabine	II	Completed	NCT00952497
FGFR	Pemigatinib	Solid tumor	Alone	II	Recruiting	NCT06653777
		UC	Alone	II	Completed	NCT02872714
		Nonmuscle invasive bladder cancer	Alone	II	Completed	NCT03914794
	Futibatinib	Solid tumors	Alone	I/II	Completed	NCT02052778
		Advanced and metastatic UC	Pembrolizumab	II	Active	NCT04601857
		Advanced cholangiocarcinoma	Alone	II	Recruiting	NCT05727176
		Advanced solid tumors	Alone	I	Completed	NCT02052778
	Rogaratinib	GIST	Alone	II	Active	NCT04595747
		Locally advanced or metastatic UC	Chemotherapy	II	Completed	NCT03410693
	Erdafitinib	Nonmuscle invasive bladder cancer	Gemcitabine, MMC	III	Completed	NCT06319820
		NSCLC, UC, GC, esophageal cancer or cholangiocarcinoma	Alone	II	Completed	NCT02699606
		UC	Midazola, metformin	II	Active	NCT02365597
β‐tubulin	Combretastatin A‐4	Advanced solid tumors	Paclitaxel, carboplatin	II	Completed	NCT00113438
		Advanced anaplastic thyroid cancer	Alone	II	Completed	NCT00060242
STING	DMXAA	NSCLC	Carboplatin, paclitaxel	I/II	Completed	NCT00832494
		Prostate cancer	Alone	II	Completed	NCT00111618
		NSCLC	Placebo, carboplatin, paclitaxel	III	Completed	NCT00662597
Phosphatidylserine	Bavituximab	NSCLC	Docetaxel	III	Completed	NCT01999673
		Breast cancer	Alone	II	Completed	NCT00669591
		Breast cancer	Paclitaxel, carboplatin	II	Completed	NCT00669565

Abbreviations: UC: urothelial cancer, PDAC: pancreatic ductal adenocarcinoma, HNSCC: head and neck squamous cell carcinoma, NSCLC: non‐small‐cell lung carcinoma, GC: gastric cancer, CEJ: cervicoesophageal junction, HCC: hepatocellular carcinoma, RCC: renal cell carcinoma, GIST: gastrointestinal stromal tumor.

*Data sources*—ClinicalTrials.gov.

#### 3.2.1 Antiangiogenic Therapy

The concept of inhibiting tumor angiogenesis was proposed over half a century ago [238]. A key advantage of antiangiogenic therapy (AAT) lies in its targeting of abnormal vascular signaling rather than directly damaging cells or inhibiting cell proliferation, as observed with radiotherapy or chemotherapy. This mechanism allows its application in chemotherapy‐resistant tumors and offers a more favorable toxicity profile compared with conventional treatments [239]. Numerous reviews have summarized the targets, agents, and clinical trials related to antiangiogenic drugs [11]. Classical antiangiogenic targets include VEGF, PDGF, and FGF (Table 2). Inhibitors against these targets have demonstrated promising antitumor efficacy in multiple clinical trials, extending patient overall survival and progression‐free survival to varying degrees. Among these, bevacizumab stands out as particularly notable. Since its approval by the United States Food and Drug Administration, bevacizumab has been utilized in various primary solid tumors and metastatic cancers, becoming a component of first‐line treatment for advanced malignancies [240, 241, 242, 243]. Other VEGF inhibitors, such as ramucirumab and olaparib, are under evaluation in multiple clinical trials and have been adopted for the treatment of specific cancers (NCT04230187, NCT03795311, NCT06121401). Ramucirumab monotherapy has shown significant overall survival benefits in HCC and advanced GC [244, 245]. Meanwhile, the addition of olaparib to bevacizumab treatment significantly prolonged progression‐free survival in ovarian cancer patients [246]. Despite the widespread clinical use of VEGF‐targeted AATs, a substantial proportion of patients develop resistance. Basic research has revealed that elevated levels of IL‐6 and FGF‐2 in obese breast cancer patients can induce resistance to anti‐VEGF therapy [247]. In epithelium‐derived malignancies, *KRAS* mutations drive the expression of angiopoietin‐2, reducing sensitivity to anti‐VEGF treatments [248]. Immune cells within the TME also contribute to AAT resistance: IL‐17 secreted by Th17 cells induces G‐CSF expression in the TME, recruiting immature myeloid cells and fostering an immunosuppressive milieu [249]. Another study showed that Treg infiltration in glioma correlates positively with AAT dosage, and post‐AAT treatment, tumors upregulate glutamate receptors leading to increased Treg accumulation [250]. These findings provide important insights for overcoming AAT resistance, suggesting the need to target initiating factors of resistance, avoid monotherapy, and combine AAT with immunotherapy or other targeted treatments. From this perspective, studies have shown that targeting CD5L—a molecule upregulated after AAT—can reverse resistance, while depleting Tregs with an anti‐CD25 antibody also restores drug sensitivity [251, 252]. Clinically, several trials combining AAT with immunotherapy are ongoing or have yet to report results (NCT03698461, NCT04017455, NCT05781308, NCT03813394). The clinical efficacy of these sensitization strategies remains to be fully elucidated.

#### 3.2.2 Vascular Disruption, Vascular Promotion, and Targeting Paracrine Signaling

In contrast to the AAT discussed earlier, which inhibits the formation of new tumor vessels, vascular disrupting agents (VDAs) target existing vascular ECs within tumors, thereby directly cutting off the blood supply to inhibit tumor growth. Agents under this therapeutic strategy can be categorized into three classes: those that destabilize microtubules in ECs, flavonoids with antivascular functions, and those targeting specific receptors on ECs [253].

The first class, comprising combretastatins, binds to the cytoskeleton of ECs, altering cell morphology, migration, adhesion, and function. A representative agent is combretastatin A4 (Table 2). The second class consists of plant‐derived flavonoids, which exhibit antitumor and vascular‐targeting effects, with vadimezan (DMXAA) being a flagship compound [254]. The third class includes agents that bind to integrins, cell adhesion molecules, or other receptors on the EC surface, inducing apoptosis [255]. In mammalian tumor models, VDAs have been shown to restore sensitivity to immune checkpoint inhibitors (ICIs) in otherwise treatment‐resistant tumors, demonstrating synergistic efficacy when combined with anti‐PD‐L1 therapy or sorafenib [256, 257, 258]. Hong et al. developed a nanomaterial‐encapsulated formulation of combretastatin A4 that through photothermal effects disrupts tumor vasculature and prevents metastasis in mouse models [259]. Meanwhile, DMXAA has been combined with digoxin, targeted therapies, or immunotherapies, as well as delivered via NP systems, to suppress the growth of various tumor types [260, 261]. Bavituximab, an antibody targeting phosphatidylserine exposed on tumor ECs, inhibits lung cancer growth in mice [262]. In clinical trials, combretastatin A4 showed short‐term improvement in patient outcomes, whereas DMXAA failed to demonstrate similar benefits [263, 264, 265]. A Phase II trial indicated that bavituximab combined with chemoradiotherapy reduced MDSC levels in GBM, and its combination with anti‐PD‐1 therapy improved the immune microenvironment in HCC [266, 267]. However, in a Phase III trial in NSCLC, bavituximab plus docetaxel (DTX) did not provide clinical benefit [268]. Therefore, although these agents show efficacy in preclinical models, their clinical application remains challenging. Factors such as drug properties and tumor type heterogeneity underline the need for larger clinical trials to validate the therapeutic potential of VDAs.

As tumors progress, dysfunctional and sparse vasculature leads to impaired blood perfusion, which severely limits the delivery of chemotherapeutics to tumor cells. Vascular promotion therapy aims to specifically address this tumor‐associated hypoperfusion. Researchers have developed a smart liposome (MC‐T‐DOX) that binds low‐dose cilengitide via an MT1‐MMP‐cleavable peptide. When reaching tumor blood vessels, MT1‐MMP—which is highly expressed in ECs—cleaves the peptide, locally releasing cilengitide [269]. This promotes EC migration and functional angiogenesis, thereby significantly increasing tumor blood perfusion [269]. Interestingly, this is not intended to nourish the tumor, but rather to reverse the state of low perfusion and create an effective delivery environment for the drug. The improved perfusion enhances the accumulation and distribution of the liposomes within the tumor; combined with thermally triggered drug release in the tumor stroma, this jointly increases drug bioavailability and therapeutic efficacy [269]. One research team coencapsulated cilengitide and DOX in liposomes for targeted release at tumor vascular endothelium, resulting in improved intratumoral blood perfusion and enhanced DOX distribution [270]. Furthermore, lysophosphatidic acid activates its receptor LPA4, promoting localized tumor vascular network formation and enhancing chemotherapeutic drug perfusion [271]. Yin et al. employed a similar strategy by codelivering NO and paclitaxel locally within tumors, increasing local drug concentration and demonstrating significant antitumor efficacy in murine models [272]. While these studies demonstrate antitumor effects in preclinical settings, the safety and efficacy of vascular promotion therapy in humans require further evaluation.

During tumor progression, the vasculature undergoes continuous remodeling. Following damage of ECs induced by radiotherapy, chemotherapy, antiangiogenic agents, or VDAs, injured blood vessels secrete angiogenesis‐related factors that paracrinally influence the local microenvironment and initiate repair processes. During this repair phase, they again release angiogenic substances, further activating ECs and establishing a “damage–repair–reactivation” positive feedback loop driven by EC‐secreted soluble Jagged‐1, initiated by vascular injury [273]. Consequently, tumor vascular ECs exhibit similar behavior under the influence of the unique TME and therapeutic pressures. This cascade of vascular‐derived secretory signals can remodel the ECM, promote tumor cell proliferation, invasion, and metastasis, induce stemness and therapy resistance, and recruit and activate immune cells, ultimately driving tumor progression and treatment failure [274]. For instance, in CRC, ECs secrete soluble Jagged‐1, which—after cleavage by ADAM17—activates the Notch pathway in adjacent tumor cells [275]. Targeting the endothelial FAK pathway can restore tumor cell sensitivity to DOX [276]. Recent studies also indicate that vascular paracrine signaling is not confined locally. EC‐derived IL‐6 can induce chemotherapy resistance in lymphoma [17]. Taylor et al. found that tumor burden hyperactivates the Notch pathway in ECs, leading to retinoic acid (RA) metabolic reprogramming that drives cachexia; this effect was blocked using RA metabolic inhibitors [277]. Current research in this field primarily focuses on elucidating the mechanisms by which vascular paracrine signaling promotes tumor progression and resistance, with relatively few studies advancing to therapeutic exploration. To date, very few clinical trials have been designed to specifically target vascular‐derived paracrine signaling pathways.

#### 3.2.3 Vascular Normalization

Two decades ago, the concept of vascular normalization was proposed [17]. Rather than being a completely novel strategy for targeting the tumor vasculature, it represents a systematic integration of antiangiogenic, vascular disrupting, vascular promotion, and vascular paracrine‐targeting strategies. The goal of vascular normalization is to control pathological angiogenesis while stabilizing existing vessels, regulating blood flow, and alleviating hypoxia [17]. As a tumor develops, it exhibits a hypoxic microenvironment alongside continuous angiogenesis. During the early phase of AAT, tumor vessels are rapidly pruned. This nutrient deprivation induces tumor apoptosis and necrosis, temporarily alleviating microenvironmental hypoxia and inducing a state of vascular normalization. However, with continued treatment, the excessive reduction of vessels leads to a return of hypoxia, which in turn drives tumor progression and contributes to resistance to antiangiogenic drugs. This phenomenon explains tumor progression in many patients treated with antiangiogenic monotherapy [277, 278]. Vascular disrupting strategies face a similar dilemma. While the destruction of tumor‐feeding vessels causes extensive tumor cell apoptosis and necrosis, the resulting severe hypoxia can spare therapy‐resistant cancer stem cells. These cells may enter a dormant state and resume proliferation under favorable conditions, ultimately leading to tumor recurrence or metastasis [279]. Vascular normalization improves the intratumoral distribution of chemotherapeutic, targeted, and immunotherapeutic agents, and enables sustained infiltration and cytotoxic activity of antitumor immune cells. Substantial basic research has identified numerous agents and targets that promote vascular normalization, including the histone deacetylase inhibitor chidamide, the small peptide JP1, the endothelial amino acid transporter inhibitor nanvuranlat, Glyoxalase 1 nano‐inhibitors, modified C‐type natriuretic peptide, and inhibition of adrenomedullin activation in TAMs [280, 281, 282, 283, 284, 285]. Furthermore, vascular normalization has been incorporated as an endpoint in several clinical trials investigating drug synergism [286, 287]. In summary, vascular normalization is a critical phase in cancer treatment. Achieving and maintaining this therapeutic window requires continuous, dynamic monitoring of the tumor. Timely integration of normalization strategies alongside other therapies holds significant potential to maximize clinical benefit for patients.

#### 3.2.4 Targeting Tumors With HEV

In recent years, intratumoral TLS have been identified and recognized as key hubs where B cells and T cells densely accumulate, driving potent antitumor immunity [288]. The density of intratumoral TLS has been strongly associated with improved response to immunotherapy and favorable patient prognosis [288]. The development and maturation of TLS depend critically on HEVs, which are specialized postcapillary venules that facilitate the recruitment of T and B cells into TLS via chemokine‐guided migration [289]. Martinet et al. first documented the presence of HEVs in human solid tumors and demonstrated their correlation with T and B cell infiltration [290]. In preclinical immunotherapy models, anti‐CTLA‐4 treatment increased intratumoral HEV density, while mouse EC antigen‐79 (MECA‐79)‐positive HEVs mediated lymphocyte migration into TLS, creating a mutually reinforcing cycle with immunotherapy [288]. Spatial transcriptomic studies of clinical specimens further revealed that IFN‐responsive HEVs promote TLS formation and are linked to favorable outcomes in nasopharyngeal carcinoma [291]. Therefore, locally inducing HEV formation within tumors has emerged as a pivotal strategy to enhance antitumor immunity. Combining antiangiogenic agents with immunotherapy can induce HEV formation in gliomas and yield promising therapeutic outcomes [292]. Mechanistically, the lymphotoxin beta receptor (LTβR) signaling pathway plays a central role in intratumoral HEV formation. LTβR agonism promotes HEV development, significantly enhances infiltration of CD8+ T cells and dendritic cells, and augments the efficacy of immunotherapy [293, 294]. Additionally, HEV formation is driven by lymphotoxin‐like, exhibiting Inducible expression, and competing with HSV glycoprotein D for HVEM, a receptor expressed by T lymphocytes (LIGHT); one research team successfully induced intratumoral TLS and overcame immunotherapy resistance using a vascular‐targeting peptide directed at LIGHT [295]. Similarly, adeno‐associated virus‐mediated LIGHT expression in brain ECs converted them into HEV‐like vessels and potentiated antiglioma immunity [296].

Based on these findings, therapies targeting the tumor vasculature have evolved from single‐target approaches toward more sophisticated and refined strategies. Targeting the tumor vasculature now necessitates distinguishing the roles of different vascular subtypes—promoting HEV formation while inhibiting other protumor vessels. Treatment must also be administered at the optimal therapeutic window and combined rationally with other modalities to achieve synergistic antitumor effects and prevent resistance. This evolution underscores the growing need for precise and dynamic monitoring of the TME.

### 3.3 Targeting CAFs

CAFs, as one of the most significant components of the tumor stroma, are deeply involved in tumorigenesis and development [297, 298]. CAFs exhibit considerable heterogeneity [297, 299, 300]. This heterogeneity is generally believed to arise partly from their diverse cellular origins. CAFs may originate from tissue‐resident fibroblasts, stellate cells, MSCs, adipocytes, ECs, chondrocytes, and smooth muscle cells [15]. Correspondingly, CAF heterogeneity manifests in significant functional diversity. Although commonly used biomarkers such as α‐smooth muscle actin (α‐SMA), FAP, and PDGFRα/β are often employed to identify CAF categories and their activation states, a limited number of unidimensional markers are insufficient for the precise delineation of CAF types and functions [301, 302, 303, 304, 305]. Notably, specific tumor types and their unique microenvironments can shape CAFs with distinct functional and gene expression profiles [304]. In studies of PDAC, based on distinct transcriptional signatures, myCAFs, which are involved in ECM formation around tumors, and iCAFs, located at the tumor periphery and capable of secreting inflammatory mediators like IL‐6, have been identified. In advanced PDAC, apCAFs with immunomodulatory capabilities have been discovered [84]. These apCAFs express MHC‐II and CD74 but lack conventional costimulatory molecules such as CD40 and CD86, which can lead to an increase in regulatory T cells and weaken antitumor immunity [306]. Furthermore, Cords et al., utilizing single‐cell and spatial proteomic data from breast cancer tissue, proposed a more general CAF classification system, categorizing CAFs into nine functional groups, including vascular CAFs, tumor‐like CAFs, IFN‐response CAFs, reticular‐like CAFs, and dividing CAFs [305]. The precise classification of CAF types and functions enhances our understanding of their complex roles and suggests that CAFs can influence tumorigenesis and development through multiple dimensions, including direct interactions with tumor cells [307, 308, 309], regulation of the ECM [310], and modulation of immune cells within the TME [301, 311].

#### 3.3.1 CAFs Depletion

**FIGURE 3:**
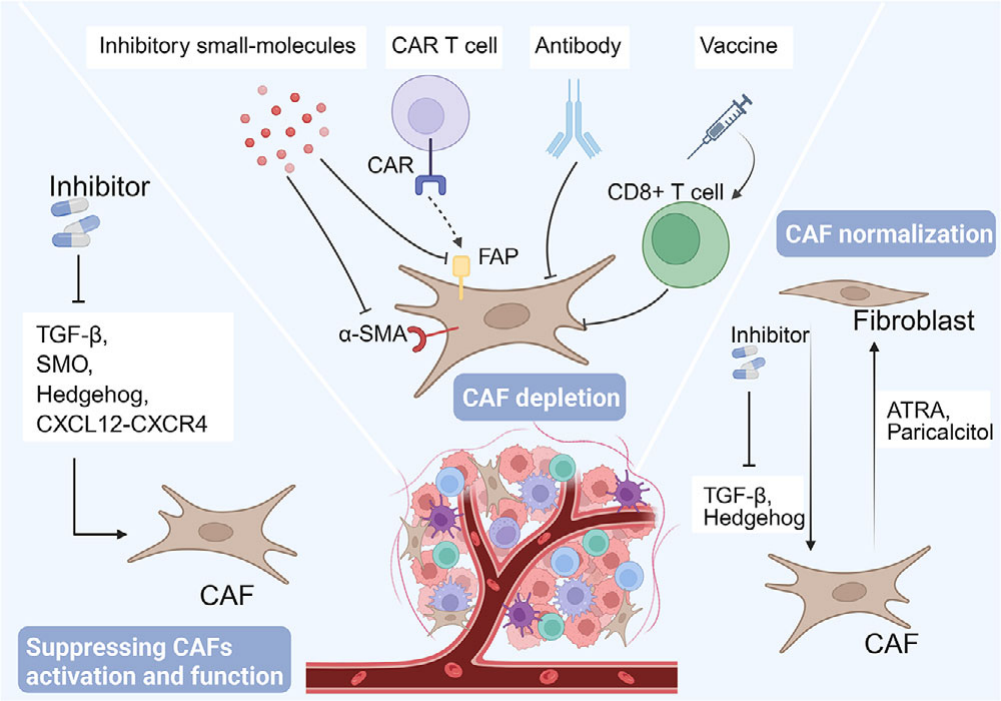
Immunotherapy strategies targeting CAFs. Three primary approaches exist for targeting CAFs and their associated molecules: (1) Directly depleting CAFs within the TME by targeting CAF markers using inhibitory small molecules or vaccines; (2) interfering with CAF activation and their regulation of the tumor ECM by inhibiting signaling pathways such as TGF‐β and Hedgehog; (3) employing ATRA and paricalcitol to reverse their activated state, shifting them from a protumor phenotype toward a more normal state. Figure 3 was created by biorender.com.

Given the extensive influence of CAFs on tumorigenesis and progression, numerous studies have been dedicated to eliminating tumor‐promoting CAFs within tumors (Figure 3). FAP is considered an ideal target for the targeted depletion of CAFs [312]. However, earlier research found that the standalone use of FAP monoclonal antibodies (sibrotuzumab) and nonselective FAP inhibitors (talabostat) failed to effectively inhibit tumor progression [313, 314]. In 2013, Jansen et al. developed the selective FAP inhibitor UAMC‐1110 [315]. Owing to its favorable selectivity and high affinity for FAP, radionuclide probes based on UAMC‐1110 have been extensively investigated [316]. Among these, diagnostic probes primarily utilize the specific binding of UAMC‐1110 to FAP to achieve imaging of CAF‐rich tumors [317]. Therapeutic probes, on the other hand, are primarily loaded with radionuclides such as ^90^Y and ^177^Lu for internal radiotherapy [318, 319, 320]. Beyond UAMC‐1110, novel FAP‐targeting small molecule‐based radioligand conjugates like FAP‐2286 (which can also be classified as a peptide–drug conjugate [PDC]) are undergoing preclinical testing [321]. Additionally, ADC drugs (OMTX705 [322]) and PDC drugs (^18^F–FAPI–42‐RGD [323], ^68^Ga–FAPI–RGD [324]) targeting FAP have been developed. However, not all of these agents function solely by depleting CAFs; they also leverage the FAP target for accurate imaging of CAF‐rich tumors and targeted delivery of antitumor drugs (exerting a bystander effect while killing FAP+ CAFs). Following the suboptimal clinical trial outcomes of FAP monoclonal antibodies [313], significant research efforts have also focused on developing antibody‐based therapies to inhibit or deplete CAFs. These include bispecific antibodies such as RO7122290 [325, 326] (targeting FAP and 4‐1BB), GEN1057 (targeting DR4, NCT06573294), and SHR‐7367 (targeting CD40, NCT05740202). Other studies have developed FAP‐targeting CAR‐T cells, dual‐target CAR‐T cells [327], and CAR‐M therapies [328, 329] to eliminate FAP+ CAFs.

#### 3.3.2 Inhibiting the Activation of CAFs and Their Regulation of the ECM

In addition to depleting CAFs, some studies focus on interfering with CAF activation and their regulatory role in the tumor ECM (Figure 3). Activation of tissue‐resident fibroblasts is a key step in CAF formation [330], with the Hedgehog [331, 332] and TGFβ [333] signaling pathways being widely reported to be involved in this activation. Small‐molecule inhibitors of the Hedgehog pathway, such as vismodegib and sonidegib [334, 335, 336], have been shown to inhibit CAF activation and exert antitumor effects. The influence of TGFβ on CAF activation is more extensive; activation of the TGFβ/SMAD pathway promotes CAF expression of αSMA and FN, enhances collagen secretion, and drives the transition of quiescent fibroblasts into myCAFs, thereby participating in ECM remodeling [84, 337]. TGFβ‐mediated CAF activation and subsequent collagen deposition also lead to reduced CD8+ T cell infiltration in the TME and diminish the efficacy of PDL1 blockade [311, 338]. Numerous drugs targeting TGFβ have been developed, including monoclonal antibodies (fresolimumab [339]), bispecific antibodies (M7824 [340]), chimeric proteins [341], and small‐molecule inhibitors (galunisertib [342]).

Beyond inhibiting CAF activation, some studies have achieved reprogramming of CAFs to reverse their activated state. Vitamin A deficiency is associated with the activation of quiescent fibroblasts, and all‐trans RA can reverse CAF activation and enhance the therapeutic effect of gemcitabine in pancreatic cancer [343, 344]. The vitamin D analogue paricalcitol has also been reported to reduce alpha‐SMA and MMP levels of/on CAFs [345]. Additionally, several studies focus on blocking the downstream effects of CAF activation, particularly their regulation of the tumor ECM. Stiffened ECM impedes the infiltration of antitumor drugs and immune cells [21, 344]. The hyaluronidase PEGPH20 was developed to degrade HA, a major component of the ECM, thereby softening the ECM [346]. LOX family enzymes secreted by CAFs are also key mediators of collagen crosslinking in the ECM [347, 348]. However, simtuzumab, an antiLOXL2 agent developed for cancer therapy, has not shown significant efficacy [198]. Small‐molecule inhibitors such as BAPN and PAT1251 are primarily used to treat organ fibrosis [349], and further investigation is needed for the development of anticancer drugs targeting this pathway.

#### 3.3.3 A Practical Framework for Integrating CAF Heterogeneity Into Therapeutic Decision‐Making

The diverse therapeutic strategies outlined above—ranging from depletion and activation inhibition to reprogramming—directly reflect the profound heterogeneity inherent to CAF populations, yet this complexity poses significant challenges for clinical decision‐making [68, 69]. To translate CAF biology into precision oncology, we propose a preliminary decision‐making framework based on CAF subset classification and biomarker profiling. The central premise of this framework is that high‐resolution analysis of tumor specimens can identify dominant functional CAF subsets and their associated biomarker signatures, thereby guiding the selection of optimal intervention strategies.

In tumors where protumorigenic CAF subsets predominate, the microenvironment is characterized by an abundance of myCAFs and iCAFs [84], accompanied by elevated levels of inflammatory mediators (e.g., IL‐6 [69], CXCL12 [101]) or ECM‐related transcripts (e.g., COL10A1 [95], LOX [347]), while apCAFs (MHC‐II+ CAFs) are comparatively scarce [68, 69]. Under these conditions, depletion or reprogramming strategies are preferred. Specifically, for FAP‐high tumors, direct elimination using FAP‐targeted CAR‐T cells, CAR‐macrophages, or FAP‐based drug conjugates represents a rational approach [312, 322, 327, 328]. For tumors exhibiting pronounced ECM deposition (a hallmark of myCAFs), inhibition of upstream activation pathways, such as TGF‐β [321, 333] or Hedgehog signaling [331, 332], can attenuate CAF function at its source. Alternatively, reprogramming activated CAFs toward a quiescent state using all‐trans RA [7] or vitamin D analogues [345] offers another effective strategy to reverse their protumorigenic phenotype.

In tumors enriched for immuno‐modulatory or tumor‐restraining CAF subsets, the biomarker landscape features high expression of MHC‐II, CD74, and PDPN, often in conjunction with TLSs [84, 85, 86, 88]. In this context, broad‐spectrum CAF depletion should be approached with caution, as targeting pan‐CAF markers such as FAP may inadvertently eliminate apCAFs or Lym‐CAFs that possess intrinsic antitumor activity, thereby compromising endogenous immune surveillance [90, 91]. A more appropriate strategy involves functional modulation combined with immunotherapy [68, 69]. For instance, leveraging the immune‐active niche fostered by these CAFs, combination with ICIs may amplify pre‐existing antitumor immune responses.

In tumors driven by discrete immunosuppressive pathways, the microenvironment is characterized by enrichment of specific CAF subsets expressing defined inhibitory molecules, such as NECTIN2+ TinCAFs [82] or Gal‐9+ CAFs [83]. For these tumors, pathway‐specific blockade offers superior precision compared with broad depletion strategies, with a reduced risk of off‐target effects. For example, when T‐cell dysfunction is primarily mediated by TinCAF‐expressed NECTIN2, targeted NECTIN2 blockade can specifically reverse T‐cell exhaustion [82]. Similarly, for immunosuppression driven by Gal‐9^hi^ CAFs, combination with Tim‐3 (the Gal‐9 receptor) inhibitors may yield synergistic benefit [82].

In summary, the future of CAF‐targeted therapy lies in moving beyond a “one‐size‐fits‐all” paradigm toward a biomarker‐guided, subset‐specific approach. By integrating multiomics data to match molecular subtype with therapeutic strategy, we can overcome the translational challenges posed by CAF heterogeneity and advance stromal targeting into the era of precision medicine.

### 3.4 Targeting TAMs

**FIGURE 4:**
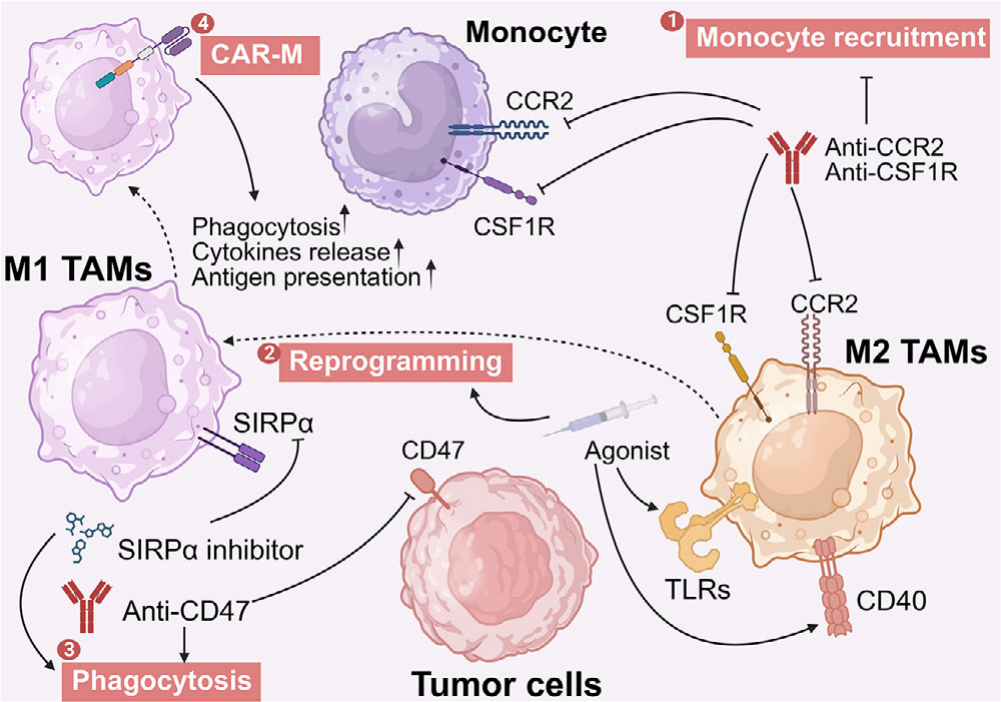
Targeting TAMs in cancer therapy. Targeting TAMs strategies can be carried out from four perspectives: (1) inhibiting recruitment of the monocyte, suppresses monocyte differentiation into macrophages; (2) remodeling M2 TAMs to become antitumor macrophages; (3) promoting phagocytic function of the macrophages; (4) remodeling TAMs to CAR‐M. Figure 4 was created by biorender.com.

Macrophages are a major class of immune cells within the TME [350]. Macrophages represent a highly heterogeneous and plastic immune cell population [351]. In terms of origin, they are divided into tissue‐resident macrophages differentiated from yolk sac erythroid‐myeloid progenitors (e.g., microglia in the brain, Kupffer cells in the liver, alveolar macrophages) [352] and infiltrating macrophages derived primarily from circulating monocytes [353, 354]. As part of the innate immune system, macrophages have long been considered a component of antitumor immunity [355]. Tissue‐resident macrophages have also been reported to inhibit distant tumor metastasis [356]. However, multiple studies have demonstrated that both tissue‐resident and infiltrating macrophages possess protumor functions [357, 358, 359, 360]. The most classic classification of TAMs categorizes them into two types: M1 macrophages, which are induced by IFN‐γ, TNF‐α, and LPS [361, 362], and M2 macrophages, which are induced by Th2 cytokines and inflammatory factors such as IL‐4, IL‐10, and TGF‐β [363]. M1 macrophages primarily inhibit tumor growth and metastasis through antibody‐dependent cellular cytotoxicity, antibody‐dependent cellular phagocytosis (ADCP), and indirect immune modulation via proinflammatory cytokines [358]. M2 macrophages mainly secrete suppressive inflammatory factors that inhibit antitumor immunity [358]. Their secreted cytokines have also been reported to promote tumor drug resistance (e.g., IL‐6) [364] and metastasis (e.g., VEGF, MMPs) [365]. With the advancement of single‐cell and spatial transcriptomic technologies, macrophage classification has been further refined, which will not be elaborated in this review. Current macrophage‐targeting therapies in tumor treatment generally aim to reduce protumor M2 macrophages and increase antitumor M1 macrophages within the TME [365] (Figure 4).

#### 3.4.1 Targeted Depletion of TAMs

Targeting the CSF1/CSF1R axis is the most direct and effective method to reduce TAM numbers, as CSF1 inhibition suppresses monocyte differentiation into macrophages at the source [366]. Current research indicates that small‐molecule inhibitors of CSF1R, such as BLZ945 [367] and PLX3397 [368], can effectively modulate tumor immunity and exert antitumor effects. There are also reports that CSF1R small‐molecule inhibitors not only deplete TAMs overall but also promote the repolarization of M2 macrophages toward the M1 phenotype [369, 370]. However, in clinical trials, blockade of the CSF1/CSF1R axis has not yielded significant patient benefit (NCT02452424, NCT03336216). Another focus has been on targeting the CCL2/CCR2 axis to inhibit TAM migration and accumulation into tumors [371]. Attempts targeting the CCL2/CCR2 axis have been largely unsuccessful. The humanized monoclonal antibody against CCL2, carlumab (CNTO 888), failed in clinical trials. CCR2 inhibitors such as RDC108 [371] and CCX872 [372] showed promising results in animal models but did not demonstrate significant antitumor activity in clinical trials [373]. Furthermore, bisphosphonates like clodronate have been found to reduce macrophages by inhibiting proliferation and inducing cell death [374, 375] and are commonly used in the treatment of bone metastases. A more precise method for targeted macrophage depletion involves the use of liposome‐encapsulated clodronate [374, 376], although related research remains at the preclinical stage.

#### 3.4.2 Reprogramming of TAMs

Due to the high plasticity of macrophages, beyond eliminating TAMs, some studies have attempted to regulate the M2/M1 macrophage ratio or reprogram TAM phenotypes to achieve therapeutic effects against tumors (Figure 4).

##### 3.4.2.1 CD47–SIRPα and CD40–CD40L Signaling Pathways

Signal regulatory protein‐alpha (SIRPα) on macrophages can bind to CD47, generating an inhibitory signal that suppresses macrophage phagocytosis. Many tumors overexpress CD47 to deliver a “don't eat me” signal to TAMs, counteracting phagocytosis [358]. Blocking the CD47–SIRPα signaling pathway has been reported in mouse models to inhibit this suppression, exert antitumor effects, and promote the repolarization of M2 macrophages toward an M1 phenotype [379]. The CD47 monoclonal antibody magrolimab (NCT04313881) was terminated early for not meeting efficacy expectations, while clinical trials for IMM01, an SIRPα‐Fc fusion protein targeting CD47 (NCT07170787), are still in their initial stages. However, these clinical trials targeting CD47 have so far been largely confined to hematologic malignancies. Activation of the CD40–CD40L signaling pathway on macrophages can upregulate MHC molecule expression and promote IL‐12 production. Activating anti‐CD40 antibodies have demonstrated tumor‐suppressive effects in various mouse tumor models [355]. CD40 agonists have also been found to enhance immune surveillance [380]. In intrahepatic cholangiocarcinoma, combination therapy with a CD40 agonist and an anti‐PD‐1 monoclonal antibody promoted the local proliferation and activation of various immune cells, including macrophages, dendritic cells, T cells, and NK cells [381].

##### 3.4.2.2 TLR Agonists

TLRs are pattern recognition receptors of the innate immune system. Their activation can promote macrophage differentiation toward a proinflammatory phenotype [382]. Local delivery of TLR agonists into tumors can facilitate TAM reprogramming toward an antitumor phenotype [383]. Specific agonists designed to mimic pathogen‐associated molecular patterns, such as acGM‐1.8 for selective TLR2 activation, have been shown to reprogram TAMs toward an M1 phenotype [384]. Among TLR agonists, only imiquimod is currently approved for clinical use, but its application in cancer therapy remains investigational and in the clinical trial phase. Resiquimod sulfate (a TLR7/8 agonist) is undergoing clinical trials for advanced melanoma (NCT06697301). TLR9 agonists, represented by CpG oligodeoxynucleotides like IMO‐2055, have been evaluated in clinical trials for various tumors including squamous cell carcinoma and clear cell renal cell carcinoma (NCT00729053, NCT01360827, NCT01040832).

#### 3.4.3 Genetic Editing and Engineering of Macrophages

In the 1980s, the Andreesen research team treated 15 patients with advanced cancer using adoptive macrophage transfer [385]. Prior to reinfusion, isolated monocytes were differentiated into macrophages and then stimulated with IFN‐γ in vitro to induce a M1 phenotype. Although primary tumors did not regress, some patients showed no significant disease progression for up to 6 months posttreatment. It has been noted that such simply “edited” macrophages might not have encountered tumor‐associated antigens, and their phenotype maintenance is subject to regulation by the TME [362]. With advancing technology, direct engineering and genetic editing of macrophages have become feasible, and CAR‐M therapy is increasingly demonstrating its therapeutic potential (Figure 4). Given the limited infectivity of conventional viral vectors (e.g., lentiviruses, retroviruses, adenovirus‐associated viruses) for macrophages, the Landau team developed a modified lentiviral vector, Vpx‐LV, capable of editing genes in myeloid cells [386]. Another option for macrophage editing is the adenovirus 5‐fiber 35 vector (Ad5f35) [387], which enables highly efficient gene editing in macrophages. Ad5f35‐infected macrophages were associated with inflammasome activation, which could promote the maintenance of an M1‐like phenotype [388]. Additionally, electroporation can also be used for gene editing and CAR‐M engineering [389]. The structure of CAR‐M is similar to that of CAR‐T, consisting of an antigen‐binding domain, a hinge region, a transmembrane domain, and one or more intracellular signaling domains [390]. A key structural difference lies in the intracellular domains: CAR‐T commonly uses CD3ζ, CD28, and 4‐1BB, whereas CAR‐M often incorporates intracellular domains such as FcRγ, MEGF10, CD86, PI3K, and TLR2/4/6 [391]. Functionally, CAR‐M primarily acts not through direct cytotoxicity like CAR‐T but by enhancing phagocytic activity and sustaining differentiation toward an antitumor phenotype [392]. The FcRγ receptor domain on CAR‐M can mediate ADCP by binding to the Fc portion of IgG antibodies [393]. Regarding target selection for CAR‐M, three targets—HER2, glypican 3 (GPC3), and mesothelin—have advanced to the clinical trial stage [394]. A HER2‐targeted CAR‐M has completed a Phase I clinical trial (NCT04660929) in solid tumors, demonstrating safety and preliminary efficacy [395]. CAR‐M therapy shows promise in solid tumors. For instance, one study engineered CAR‐M with an intracellular CD147 domain to increase macrophage MMP expression, thereby enhancing CAR‐M‐mediated disruption of the tumor basement membrane and improving infiltration [396]. This enhanced solid tumor penetration did not compromise their phagocytic activity, inflammatory cytokine production, or reactive oxygen species (ROS) generation. Given these capabilities, CAR‐M holds significant potential for the treatment of solid tumors.

### 3.5 Targeting ECs

ECs primarily mediate blood–tumor barrier permeability and tumor angiogenesis within the TME [397]. Tumor‐associated ECs regulate the entry of molecules and cells into the TME, interact with immune cells, and function as hapten‐presenting cells to promote immune cell activation and proliferation, thereby playing a crucial role in antitumor immune regulation [398]. Simultaneously, they contribute to the formation of TLSs, thereby enhancing the efficacy of ICIs [399]. Compared with normal ECs, tumor‐associated ECs exhibit phenotypic, gene expression, and functional abnormalities, possessing high proliferative potential and activating immune suppression mechanisms to support tumor progression and metastasis. Consequently, an emerging therapeutic strategy combines AAT with immunotherapies (such as ICIs, CAR‐T, and bispecific antibodies) to simultaneously target ECs and immune cells—blocking angiogenesis while enhancing CD8+ T cell recruitment and activation.

AATs targeting vascular endothelium primarily achieve vascular normalization by inhibiting tumor angiogenesis and reversing abnormal tumor‐associated EC phenotypes (e.g., hyperproliferation and hyperpermeability) [400]. Mechanisms include reducing vascular leakage to alleviate interstitial pressure, improving perfusion and oxygenation to enhance drug delivery, and restoring endothelial responsiveness to inflammatory signals to promote effector T cell infiltration [401, 402]. Representative agents like the anti‐VEGF antibody bevacizumab and the VEGFR tyrosine kinase inhibitor crizotinib have demonstrated efficacy in cancers such as colorectal carcinoma and GBM [403, 404, 405]. Beyond VEGFR2, VEGF‐A and its receptors VEGFR1, VEGFR3, as well as VEGF‐C, represent critical targets. For instance, the signaling pathway involving VEGFR3 and its ligand VEGF‐C participates in lymphatic angiogenesis, promoting tumor lymphatic metastasis. Related inhibitors (such as sorafenib and sunitinib) are already in clinical use [406]. PDGF and its receptors participate in perivascular cell recruitment, influencing vascular maturation, and support tumor cell growth and dissemination by providing routes for intravasation and hematogenous spread to distant organs [407]. Adhesion molecules like ICAM‐1, VCAM‐1, and CLEVER‐1 regulate immune cell migration to influence tumor progression. For instance, the anti‐CLEVER‐1 antibody beramulimab reduces Treg cell infiltration and inhibits tumor progression [408].

In recent years, targeted EC therapy combined with ICIs has garnered significant attention. The core mechanism of this combination approach involves improving tumor vascular abnormalities through antiangiogenic treatment while simultaneously lifting immune suppression via ICIs, creating a positive feedback loop of “vascular normalization‐immune activation.” Antiangiogenic agents (e.g., anti‐VEGFR2 antibodies, bevacizumab) normalize disrupted tumor vasculature, enhance drug delivery efficiency, and reverse endothelial “nonresponsiveness” while upregulating ICAM‐1, VCAM‐1, thereby promoting effector T cell infiltration. Meanwhile, ICIs (such as anti‐PD‐1/PD‐L1 and anti‐CTLA‐4 antibodies) block immune checkpoint pathways to activate T cell function. The Th1‐type immune responses they induce (e.g., IFN‐γ secretion) further promote vascular normalization and enhance immune cell recruitment, forming a positive feedback loop [403, 409]. Targeting tumor‐associated ECs low‐dose anti‐VEGFR2 antibodies can upregulate PD‐1 level on immune cell surfaces by stimulating osteopontin and TGF‐β secretion, thereby sensitizing breast cancer to a PD‐1 blockade. Combining anti‐VEGFR2 with anti‐PD‐L1 induces HEV formation in pancreatic and breast cancer models, synergistically promoting T cell infiltration and activation.

Multiple clinical trials have validated the efficacy and safety of combining antiangiogenesis with immunotherapy across various tumors. The combination of bevacizumab and the anti‐CTLA‐4 antibody ipilimumab reverses EC nonresponsiveness, increases adhesion molecule expression, thereby improving immune cell recruitment and clinical prognosis in melanoma patients. The combination of bevacizumab and the anti‐PD‐L1 antibody atezolizumab demonstrates promising antitumor activity in renal cell carcinoma, HCC, and NSCLC. Novel bispecific antibodies (such as HB0025 and AK112, which simultaneously block PD‐L1/VEGF) have also entered clinical trials, showing potential advantages [410]. Furthermore, the combination of the anti‐PD‐1 antibody pembrolizumab with the multitargeted tyrosine kinase inhibitor cabozantinib significantly improved progression‐free survival and overall survival in HNSCC, correlating with baseline CD8^+^ T‐cell infiltration levels [411]. The recent Phase II BREAKPOINT trial further confirmed cabozantinib's potential as a follow‐up therapy after ICI treatment in renal cell carcinoma patients [412]. These findings support the combination of antiangiogenesis and immunotherapy as a promising strategy to enhance efficacy against refractory tumors.

While combination therapy with antiangiogenic agents and ICIs demonstrates significant efficacy, it still faces challenges including dose management, adverse effects, and patient stratification. First, the dosage, sequence, and timing of different drugs significantly impact efficacy. Excessive doses of antiangiogenic agents can cause vascular injury, hypoxia, and immunosuppression, paradoxically inducing resistance to the combined therapy of ICIs and antiangiogenic agents [413]. Second, common adverse reactions include dose‐dependent hypertension, thrombocytopenia, proteinuria, hepatotoxicity, and nonspecific immune system activation. Finally, combination therapy is not suitable for all patients, particularly those with autoimmune diseases, organ transplant history, elderly individuals, or those at risk of bleeding or with severe cardiovascular, hepatic, or gastrointestinal diseases, as they are more prone to serious complications [414]. Therefore, optimizing treatment regimens and selecting appropriate patient populations are critical to realizing the clinical value of combination therapy.

### 3.6 Targeting MSCs

#### 3.6.1 Depletion of Tumor‐Associated MSCs

**FIGURE 5:**
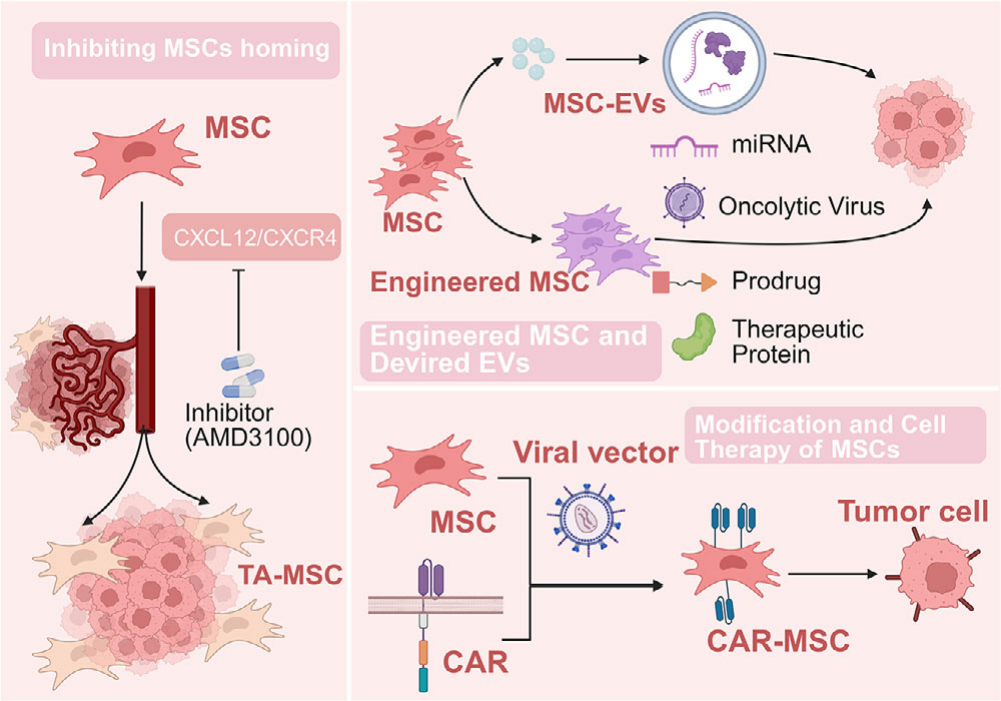
Targeting tumor‐associated mesenchymal stem cells (MSCs) and their application in cancer therapy. MSCs recruited by the tumor microenvironment via the CXCL12/CXCR4 signaling pathway are converted into TA‐MSCs, a process that can be blocked by CXCR4 inhibitors such as AMD3100. MSCs themselves or their released EVs can serve as drug delivery platforms, loading various therapeutic agents including chemotherapeutic drugs, therapeutic proteins, miRNAs, oncolytic viruses, and more, leveraging their tumor homing capacity to target tumor sites. MSCs can be modified using tools like viral vectors, for example, to express chimeric antigen receptors (CARs), transforming them into CAR‐MSCs. These genetically engineered MSCs can directly target and attack tumor cells, enabling precise cell‐based therapy. Figure 5 was created by biorender.com.

Given the protumor effects of tumor‐associated MSCs (TA‐MSCs), one strategy to eliminate TA‐MSCs involves targeting the classic CXCL12/CXCR4 signaling pathway, which mediates the tumor‐homing ability of MSCs [415] (Figure 5). Numerous attempts have been made to block the CXCL12/CXCR4 axis. In animal experiments, the CXCR4 inhibitor AMD3100 [416] and a high‐affinity anti‐CXCL12 PEGylated mirror‐image l‐oligonucleotide (olaptesed–pegol) [417] have demonstrated certain therapeutic effects. However, these studies were not specifically directed at TA‐MSCs and their CXCL12/CXCR4 signaling pathway. Currently, there is still a lack of in‐depth research on targeted clearance of TA‐MSCs.

#### 3.6.2 Utilizing MSCs and Their Exosomes as Delivery Carriers

Leveraging the tumor‐homing property of MSCs, some studies have attempted to use MSCs or their exosomes as carriers to deliver drugs or for therapeutic modifications (Figure 5). A major drawback of chemotherapy drugs is their lack of precise targeting to tumors. MSCs possess a well‐documented tumor‐homing capacity, primarily driven by inflammatory signals and chemokines (e.g., SDF‐1α, CCL2) secreted by the TME, which actively recruit MSCs to tumor sites [184]. This intrinsic tropism makes them attractive candidates as living drug delivery vehicles. For example, MSCs loaded with paclitaxel and engineered MSCs carrying DOX have shown promising therapeutic effects in mouse models by leveraging this homing mechanism to deliver chemotherapeutics directly to tumors [418]. Furthermore, studies have found that cryopreserved MSCs not only maintain therapeutic efficacy and tumor‐targeting capability but also exhibit higher drug‐loading capacity [419]. In addition to drug delivery, MSCs can also serve as carriers for oncolytic viruses [420, 421], or as gene editing vectors [422] for tumor treatment.

Similar to MSCs, MSC‐derived EVs (MSC‐EVs) also possess tumor‐targeting capabilities [423]. Numerous studies have demonstrated that MSC‐EVs can deliver various cargoes, including chemotherapy drugs, proteins, and RNA. For instance, MSC‐EVs have been used to codeliver gemcitabine and paclitaxel [424], deliver the noncoding RNA miR‐503‐3p [425], and deliver the TRAIL protein (tumor necrosis factor‐related apoptosis‐inducing ligand) [426].

#### 3.6.3 Modification and Cell Therapy of MSCs

Early attempts to modify MSCs were represented by their use to overexpress IFN‐β [427]. Subsequently, many studies have explored using MSCs to deliver antitumor cytokines such as IL‐2, IL‐7, IL‐12, IL‐15, and IFN‐γ to tumor sites [428, 429, 430]. Similarly, there have been attempts to engineer MSCs to express specific antibodies for cell therapy. For example, Aliperta et al. developed MSCs expressing a CD33–CD3 bispecific antibody, which demonstrated excellent antitumor effects in a mouse AML model [431].

Regarding MSC modification, recent attempts have also been made to engineer MSCs to express CARs [432]. Compared with traditional CAR‐T therapy, CAR‐MSCs offer better targeting and favorable safety profiles. CAR‐MSCs retain the negative immunomodulatory properties of MSCs, and there have been attempts to use them for treating graft‐versus‐host disease [433]. Researchers have genetically modified MSCs to target E‐cadherin, a key protein in T‐cell activation, for the treatment of graft‐versus‐host disease [432]. In antitumor therapy, related research also exists. Golinelli et al. constructed CAR‐MSCs capable of simultaneously expressing TRAIL and an anti‐GD2 CAR, which could target and eliminate GD2‐positive GBM [434].

#### 3.6.4 Safety and Clinical Translation Considerations

Despite the considerable promise of MSCs and MSC‐EVs as therapeutic vehicles, their clinical translation faces multiple challenges, warranting some caution. First and foremost, safety is a paramount concern. MSCs possess a context‐dependent dual functionality, and within the TME, they risk being “educated” or polarized into a protumorigenic phenotype, potentially counteracting therapeutic benefits or even promoting disease progression [109, 111, 112]. For instance, MSC‐EVs from certain sources may promote rather than inhibit tumor cell proliferation [119, 120]. Therefore, rigorous screening of MSC source, donor, and culture batch, coupled with genetic engineering to enhance therapeutic specificity and suppress protumor potential, is a critical prerequisite for ensuring safety.

In addition, standardization of manufacturing processes and quality control presents a significant bottleneck. MSCs exhibit considerable heterogeneity between donors and tissue sources, and in vitro expansion can lead to cellular senescence, phenotypic drift, and functional loss [435]. Similarly, standardized protocols for the isolation, purification, characterization, and drug‐loading of MSC‐EVs are lacking [435]. Batch‐to‐batch variations in yield, purity, size distribution, surface markers, and drug‐loading efficiency directly impact the reproducibility of product efficacy and safety.

Furthermore, the in vivo biodistribution and long‐term fate of these carriers remain inadequately defined. Although MSCs possess tumor‐homing capacity, a significant proportion of systemically administered cells may lodge in organs such as the lungs and liver [436]. The current absence of highly sensitive, long‐term, noninvasive in vivo tracking technologies to precisely monitor the real‐time distribution, survival, differentiation, and ultimate clearance of carrier cells poses a major obstacle for assessing off‐target effects and long‐term toxicity [436].

The regulatory pathway is complex. MSC and MSC‐EV products fall under the category of advanced therapy medicinal products or complex biologics, subject to stringent regulatory requirements [435]. This necessitates establishing good manufacturing practice‐compliant production processes, completing comprehensive nonclinical safety evaluations (including tumorigenicity, immunogenicity, distribution, and metabolism), and designing rigorous clinical trials to demonstrate a favorable risk‐benefit profile compared with conventional therapies.

### 3.7 Tumor Pericytes

Pericytes are widely distributed within the tumor stroma. Known for maintaining the blood–brain barrier, participating in capillary formation, and regulating capillary blood flow, they also possess the potential to differentiate into ECs and fibroblasts [437]. In the TME, pericytes actively contribute to tumor progression [438]. These characteristics make them attractive therapeutic targets for modulating vascular normalization and reducing the pool of CAFs. In glioma, pericyte‐targeting strategies have been explored relatively early. Ibrutinib was shown to improve blood–brain barrier permeability and enhance chemotherapy efficacy, prolonging survival in animal models [437]. Similarly, a pericyte‐targeting prodrug restored tumor sensitivity to AAT in mice [439, 440]. Studies have also revealed that hexokinase 2‐driven glycolysis is upregulated in tumor pericytes; inhibition with 3‐bromopyruvate disrupts this metabolic pathway and suppresses pericyte function [441]. CD248, expressed on the pericyte surface, represents another potential target. Treatment with a β‐catenin inhibitor downregulated CD248 expression in lung cancer pericytes and suppressed tumor angiogenesis [442]. However, the anti‐CD248 monoclonal antibody ontuxizumab failed to demonstrate efficacy in a Phase II trial, indicating the need for more refined targeting approaches [443]. As an alternative, Ash et al. developed CD248‐targeting CAR‐T cells, which effectively suppressed tumor growth across multiple immunocompetent mouse models, showing significant clinical potential [444]. Other innovative strategies include an alpha‐Type I polarized dendritic cell vaccine and a *Listeria*‐based vaccine targeting the pericyte antigens DLK1 and RGS5, respectively, both of which inhibited CRC progression in preclinical models [445, 446]. Recent research highlights PDGFRβ‐positive pericytes as key contributors to ECM formation. The selective PDGFR inhibitor CP‐673451 significantly suppressed pericyte differentiation and tumor growth in preclinical models. Additionally, targeting GPR91 on PDGFRβ+ pericytes restored tumor sensitivity to tyrosine kinase inhibitors [447, 448]. Zhang et al. reported that irradiated tumor cells secrete plasminogen activator inhibitor‐1 (PAI‐1), inducing pericytes to differentiate into secreted frizzled‐related protein 2 (SFRP2)‐high CAFs. Blocking either PAI‐1 or SFRP2 inhibited the abscopal effect following radiotherapy [449]. Furthermore, low‐dose combretastatin A4 and eribulin were found to selectively bind pericyte tubulin and inhibit pericyte differentiation, thereby promoting vascular normalization. These findings provide guidance for the precise clinical application of these agents [450]. Collectively, these findings underscore the importance and targetability of pericytes in the tumor stroma. However, our understanding of their tumor‐promoting mechanisms remains incomplete. Further elucidation of pericyte functions and subsets will pave the way for novel therapeutic strategies that exploit pericytes as central hubs for cancer treatment, potentially benefiting more patients in the future.

## 4 Nanomaterials for Targeting ECM in Cancer Therapy

The formidable barriers presented by the dense and complex tumor stroma, particularly the ECM, significantly limit the delivery and efficacy of conventional therapeutics. To overcome these challenges, the field has turned to engineered nanomaterial‐based delivery systems. This chapter is dedicated to reviewing these innovative platforms designed to target and remodel the tumor stroma. We focus on the application of various nanomaterials—including liposomes, polymeric NPs, dendrimers, and metal–organic frameworks (MOFs)—that are engineered for enhanced drug penetration and accumulation within the stromal matrix (Table 3). Key design strategies are discussed, such as the codelivery of stroma‐modulating agents (e.g., hyaluronidase, collagenase, PFD) with cytotoxic drugs, the incorporation of stimuli‐responsive elements (e.g., pH, enzymes, near‐infrared [NIR] light) for controlled drug release, and surface modifications for active targeting. The discussion is supported by a summary of preclinical studies across different cancer models, illustrating how these smart nanocarriers can degrade ECM components, normalize tumor vasculature, reverse immunosuppression, and ultimately potentiate the antitumor effects of their therapeutic cargo. This section highlights the convergence of nanomedicine and stromal targeting as a promising frontier for improving cancer therapy.

**TABLE 3:** Applications of nanomaterials in targeting tumor stroma in cancer treatment.

Nanomaterial	Component	Reagent	Stimulus–response	Cancer model	References
Liposome	DSPE–PEG‐modified liposomes	SREKA targeting peptide	Not explicitly specified	Highly metastatic breast cancer	[451]
	Thermosensitive liposomes (TSL) coloading ultrasmall melanin nanoparticles (MNP) and DOX	Near‐infrared (NIR) light triggering size transformation	NIR laser‐induced photothermal effect causes liposome rupture and release of MNP/DOX	4T1 breast cancer	[452]
	Self‐targeting DCPA liposomes loaded with gemcitabine, bromelain, and PEGylated ciprofloxacin	Bromelain	pH‐responsive (liposome disruption in the acidic TME)	Pancreatic cancer	[453]
	Glycyrrhetinic acid (GA)‐conjugated liposomes codelivering doxorubicin and berberine	Berberine	Active targeting (via GA receptors overexpressed on cancer cells)	HCC	[454]
	Biomimetic liposomes with surface‐bound neutrophil elastase (NE)	Neutrophil elastase	Enzyme‐mediated degradation of elastin and collagen in the ECM	TNBC	[455]
	PEGylated liposomes loaded with zoledronic acid, combined with candesartan	Candesartan	Vascular normalization (via inhibition of the TGF‐β1 pathway)	4T1 mouse breast cancer	[456]
	Membrane‐fusion liposomes (TAFsomes load pirfenidone, CCMsomes load doxorubicin)	Pirfenidone	Sequential release (initial targeting of tumor‐associated fibroblasts, followed by cancer cell targeting)	TNBC	[457]
	Hyaluronidase (HAase)‐conjugated quercetin liposomes	Hyaluronidase	HAase‐mediated ECM degradation	Pancreatic cancer	[458]
	Liposomes encapsulating collagenase Type I	Collagenase	Controlled enzymatic release (targeted degradation of collagen)	PDAC	[459]
	MMP‐2 responsive peptide–hybrid liposomes loaded with pirfenidone (PFD)	Pirfenidone	MMP‐2 enzyme‐responsive drug release (activated in the tumor stroma)	Pancreatic cancer	[460]
	Thermo‐responsive liposomes loading FeS_2_ and cGAMP, surface‐modified with bromelain	Bromelain	NIR‐II light‐controlled mild photothermal effect triggers drug release	4T1 breast cancer model	[461]
Polymer micelles	PArg, DSPE–PEG, TPP–PEG	CPT, CA, NLG919	ROS‐responsive; NO release activates MMPs for ECM degradation	TNBC	[462]
	P(HPMA‐co‐MAA)‐b‐PMMA block copolymer	Doxorubicin	/	Pancreatic multicellular tumor spheroids	
	HA, Se‐Se bond‐modified polymer	Valsartan, Doxorubicin	FAP‐α enzyme‐responsive	TME (specific model not specified)	
	PEG–PBLG polymer	Tranilast	/	Mouse breast cancer	[465]
	PEG–pArg–pLys–NI polymer, HA coating	p‐GEM, silybin	Hypoxia‐responsive; CD44 targeting	PDAC	[466]
	CSOSA, Tel conjugation	Doxorubicin	AT1R targeting	Breast cancer	[467]
	mPEG–hyd–PLGA polymer	Paclitaxel, AMD3100, BMS‐1	pH‐responsive	TNBC	[468]
	PEG–PLA polymer, CREKA peptide modification	α‐Mangostin	CAF targeting	PANC	[469]
	mPEG–PDPA–PG polymer, HAase coating	Epirubicin	pH‐responsive; HAase degrades HA in ECM	Tumor spheroid model	[470]
Polymeric NP	HPMA copolymer–CHP conjugate	Not specified (targeted delivery)	EPR effect; collagen targeting	MDA‐MB‐231 breast cancer	[471]
	Semiconductor polymer (PCB‐N3)/PEG–CB	BAPN	Cathepsin B enzymatic	4T1 breast cancer	[191]
	Hyperbranched polyglycerol (HPG) with PBA masking	Batimastat, doxorubicin, BMS‐1	pH/ROS responsive	4T1 breast cancer, CT26 colon cancer	[472]
	mPEG–PLGA copolymer	Baicalein	Passive targeting (EPR)	TNBC mouse model	[473]
	Dendritic POEGMA polymer	Dasatinib, epirubicin	Cathepsin B/pH responsive	4T1, MDA‐MB‐231 breast cancer	[474]
	mPEG–PLLys (BZD) copolymer	Gemcitabine, sulfopin	pH responsive (pH 6.5)	Pancreatic cancer	[475]
	HES–FA conjugate	IR780, DOX–SS–DOX prodrug	Reductive (GSH)	TNBC model	[475]
	AEAA–PEG–PCL copolymer	IPI‐549, silibinin	Sigma receptor targeting	Breast cancer model	[476]
	PLGA nanoparticles	Doxorubicin, metformin, losartan	EPR	B16F10 melanoma	[477]
Carbon‐based nanomaterials	y‐CDCs, CCM coating	DOX	Redox (GSH)	B16 melanoma	[478]
	GFNPs	GEM	/	PDAC	[479]
	HMCNs, iRGD‐TSLs	Losartan	Photothermal (NIR laser)	4T1 breast cancer	[480]
	CDs, peptide crosslinked	DOX, Fe ions, losartan	CAF‐responsive (FAP‐α)	4T1 breast cancer	[481]
	CDs	SN38	pH‐responsive	CRC	[482]

### 4.1 Liposomes

Liposomes are nanoscale vesicles composed of phospholipid bilayers, featuring an internal aqueous core and a hydrophobic membrane structure [483]. Owing to their excellent biocompatibility, modifiability, and drug delivery capacity, nanoliposomes are emerging as promising carriers for targeting ECM [19] (Table 3). Among the extensively investigated targeting strategies, surface modification or encapsulation of ECM‐degrading enzymes (e.g., collagenase, hyaluronidase, elastase) onto or within liposomes has been widely adopted to disrupt the tumor ECM barrier and enhance drug penetration [455, 459, 460]. However, free enzymes are prone to inactivation in vivo and may cause nonspecific degradation of normal tissue ECM. For instance, although collagenase‐I can reduce IFP, systemic administration carries potential risks of tissue damage [484]. To address the instability of free enzymes, Zhou et al. conjugated recombinant human hyaluronidase PH20 (rHuPH20) with PLGA–PEG copolymers and further coated the complex with a low‐density PEG layer, which significantly prolonged the enzyme's circulation half‐life [485]. Another strategy involves encapsulating NPs with cell membranes (e.g., erythrocyte membrane, macrophage membrane) to improve biocompatibility and targeting specificity [486]. For example, platelet membrane‐coated MOFs can specifically recognize collagen secreted by CAFs, enabling precise drug delivery [487]. To achieve targeted enzyme release at the tumor site while minimizing systemic toxicity, environmentally responsive liposomes have been developed that undergo structural changes or drug release in response to TME‐specific signals. For instance, Ji et al. designed MMP‐2‐responsive peptide–hybrid liposomes that release the antifibrotic drug PFD in MMP‐2‐overexpressing tumors, thereby reducing ECM deposition [460]. Similarly, Li's team developed pH‐sensitive NPs coloaded with bromelain and DOX, which rapidly release bromelain in the weakly acidic TME to degrade ECM locally and enhance DOX penetration [488].

Limited by high tumor IFP and abnormal vasculature, nanoliposomes face challenges of restricted drug penetration [489]. Researchers have employed liposomes loaded with NO donors to indirectly degrade the ECM via MMP activation, while improving vascular permeability and enhancing the intratumoral distribution of gemcitabine‐loaded liposomes [490, 491]. Wang et al. developed a NIR‐responsive thermosensitive liposome that undergoes phase transition upon NIR irradiation, releasing small‐sized DOX (∼10 nm) to penetrate dense ECM and simultaneously triggering the synergistic release of ICIs [492]. Furthermore, Hu et al. utilized a VEGFR inhibitor (bevacizumab) in combination with ECM‐degrading liposomes, achieving a 3.2‐fold increase in tumor penetration depth and a 4.5‐fold enhancement in CD8^+^ T‐cell infiltration in the combination therapy group [493].

Studies have demonstrated that ECM fragments may mimic chemokines to attract inflammatory cells, thereby accelerating tumor progression [494, 495]. Consequently, researchers have shifted their focus to ECM synthesis intervention strategies. For instance, small‐molecule inhibitors such as 4‐methylumbelliferone have been utilized to reduce HA synthesis [496, 497]. Additionally, nanoliposome‐mediated delivery of prolyl‐4‐hydroxylase (P4H) inhibitors (e.g., sh‐P4HA2, 1,4‐DPCA) has been employed to inhibit collagen hydroxylation and stabilization [498, 499, 500, 501]. However, ECM synthesis inhibition requires prolonged administration (e.g., LOS necessitates continuous dosing for at least 7 days at ≥40 mg/kg/day), and the alignment between the sustained‐release properties of nanoliposomes and the “effective time window” remains suboptimal [502]. Furthermore, coloading multiple inhibitors (e.g., all‐trans RA (ATRA) + siHSP47) or incorporating targeting ligands increases the complexity of nanoliposome design and poses challenges for scalable production [503].

NPs targeting CAFs, particularly nanoliposomes, have garnered extensive research attention in recent years. For instance, glycyrrhetinic acid (GA)‐modified liposomes (Dox&Ber@lip‐GA) target hepatic CAFs via GA receptors, significantly enhancing drug delivery efficiency [454]. Peptides targeting FAP‐α and urokinase plasminogen activator receptor have been demonstrated to improve the targeting specificity of chemotherapeutic agents across various cancer models [464, 493, 504, 505, 506]. Furthermore, liposomes loaded with TGF‐β inhibitors (e.g., LY2109761, tranilast) or antifibrotic drugs (e.g., PFD) effectively inhibit CAF activation and reduce ECM synthesis [460, 507]. Delivery of vitamin D receptor ligands (e.g., calcipotriol) or relaxin‐2 via liposomes can reprogram tumor‐promoting CAFs into a quiescent state, thereby improving drug penetration [508, 509]. However, the high heterogeneity of CAFs limits the efficacy of single‐target approaches (e.g., FAP‐α), which may fail to cover all CAF subtypes and risk damaging normal fibroblasts [330, 510]. To address this, Zang et al. developed an innovative dual‐targeting strategy using hybrid membrane‐coated solid lipid NPs. They fused membranes from breast cancer cells (4T1) with those from TGF‐β1‐activated NIH3T3 fibroblasts (a widely used in vitro model for CAFs) to create the NPs. While NIH3T3 is an immortalized mouse embryonic fibroblast cell line and thus differs from primary, nonimmortalized CAFs found in vivo, this model successfully recapitulates key activation markers (e.g., FAP‐α, α‐SMA). The resulting biomimetic NPs demonstrated significantly enhanced dual‐targeting precision toward both cancer cells and the CAF model in vitro and in vivo [511]. Similarly, Jia et al. designed membrane‐fusogenic liposomes that separately target CAFs and tumor cells by leveraging the innate homing properties of their respective membranes. This system enables asynchronous delivery of the antifibrotic agent PFD and the chemotherapeutic drug DOX, effectively remodeling the TME [457]. While biomimetic coatings partially overcome heterogeneity‐related challenges, the processes of membrane extraction and purification require further optimization.

### 4.2 Polymer Micelles

Polymeric micelles are colloidal aggregates formed by the self‐assembly of amphiphilic block copolymers in aqueous solution (Table 3). When the concentration of these amphiphilic polymers exceeds the critical micelle concentration (CMC), they spontaneously assemble into micellar structures; below the CMC, the polymers exist as unimers [512]. These nanostructures typically exhibit a core–shell architecture: hydrophobic polymer segments comprise the inner core, serving as a reservoir for poorly soluble drugs, while hydrophilic segments form the outer shell, which confers colloidal stability, prevents aggregation, and minimizes nonspecific protein adsorption in biological environments [513]. Poly(ethylene glycol)–poly(lactic acid) (PEG–PLA) and its copolymer derivative PEG–poly(lactic‐co‐glycolic acid) (PEG–PLGA) represent one of the earliest and most extensively investigated classes of micellar scaffold materials, prized for their tunable biodegradability, excellent biocompatibility, and well‐defined molecular structures [469]. For instance, Duan and colleagues employed PEG_2000_–PLA_1300_‐based micelles to encapsulate paclitaxel. The hydrophobic core enabled efficient loading of the lipophilic drug, while the hydrophilic PEG corona provided a steric barrier that reduced plasma protein adsorption, prolonged systemic circulation, and consequently enhanced tumor‐specific drug accumulation via the enhanced permeability and retention effect [514].

Conventional micelles, stabilized primarily by weak intermolecular interactions such as hydrophobic forces, face significant challenges in vivo, including disassembly risks from protein adsorption and dilution below the CMC [513]. Studies demonstrate that uncrosslinked micelles (UCM) exhibit poor stability under physiological conditions. For example, DOX release from UCM reached 83.3% within 2 h, substantially higher than the 25.8% release from crosslinked micelles (CKM) at pH 7.4. This premature drug leakage not only diminishes therapeutic efficacy but also increases systemic toxicity [463]. To address the instability of conventional micelles, chemical crosslinking strategies have been employed to reinforce their structure. Micelles composed of poly(N‐(2‐hydroxypropyl)methacrylamide‐co‐methacrylic acid)‐block‐poly(methyl methacrylate) (P(HPMA‐co‐MAA)‐b‐PMMA), for example, were stabilized via covalent crosslinking of shell carboxyl groups with 1,8‐diaminooctane, forming CKM [463]. These CKM demonstrated significantly superior penetration and drug delivery efficacy compared with UCM in pancreatic multicellular tumor spheroid models [463]. However, excessive crosslinking can impede drug release. CKM released only 69.8% of DOX over 48 h at pH 5.0, a release kinetics potentially too slow for effective tumor therapy. Similarly, Suzuki et al. reported cationic micelles with a highly hydrophobic core exhibiting ultra‐slow release (13% DOX over 72 h), where excessive stability might compromise intracellular drug bioavailability [515]. Furthermore, Panagi et al. highlighted that micelle dilution below the CMC (8 µg/mL) causes rapid drug leakage, whereas concentrations above the CMC significantly restrict release, revealing a narrow therapeutic concentration window [465].

To resolve this stability‐release dilemma, stimulus‐responsive micelles have been developed. Zhang et al. designed pH‐sensitive micelles based on hydrazone bonds, which released only 23.1% at neutral pH (ensuring circulatory stability) but 58.7% in the acidic TME (pH 5.0), achieving an optimal balance [468]. Enzyme‐responsive micelles utilize substrates cleavable by tumor‐associated enzymes, such as MMPs. Micelles labeled with the MMP‐2‐sensitive peptide GPLGIAGQ showed a 3.2‐fold higher drug release at tumor sites compared with normal tissues [515]. To combat tumor heterogeneity, surface‐functionalized micelles enable multitargeting synergy. cRGD‐modified methoxy PEG (mPEG)–PDLLA micelles enhance blood–brain barrier penetration by targeting overexpressed integrins on glioma cells and tumor vasculature, leading to 4.7‐fold higher tumor accumulation compared with nontargeted counterparts in a glioma model [465]. Li et al. developed an intelligent MP@HA micelle system that modulates surface charge for enhanced uptake. HA‐coated micelles, upon hyaluronidase exposure, shifted zeta potential from −15.3 to +5.8 mV, increasing cellular internalization by 3.1‐fold [466]. Dynamic structural designs also improve adaptation to delivery barriers. Thermosensitive Pluronic P105 micelles dissociated from 50 nm micelles to 8 nm unimers at 41°C, increasing penetration depth in multicellular layers by 2.8‐fold [463]. Recently, Ma et al. constructed a theranostic system by covalently conjugating HA with adipic dihydrazide (ADH) and the NIR dye IR808. This micelle leverages HA–CD44 receptor targeting for active delivery and utilizes ADH for chemical exchange saturation transfer MRI contrast (signals at 4.4 and 5.4 ppm), enabling precise intratumoral localization of the photosensitizer [516]. Future directions emphasize developing more precise enzyme‐responsive materials, constructing modular “all‐in‐one” platforms, and integrating imaging‐guided theranostic systems.

### 4.3 Polymeric NPs

Polymeric NPs are particles ranging in size from 1 to 1000 nm, composed of polymeric materials, and can be categorized into nanocapsules and nanospheres based on their morphology [517, 518]. Nanocapsules possess an oily core (typically for drug dissolution) and a polymeric shell, enabling controlled drug release. Nanospheres consist of a continuous polymeric network where drugs can be embedded within the matrix or adsorbed onto the surface [519]. These particles are generally formulated from synthetic or natural polymers and demonstrate considerable potential in drug delivery, offering capabilities such as controlled release, protection of drug activity, enhanced bioavailability, and improved therapeutic index [520]. Preparation methods for polymeric NPs primarily fall into two strategies: dispersion of preformed polymers or polymerization of monomers. Commonly employed techniques include solvent evaporation (forming nanospheres or nanocapsules via emulsification, solvent diffusion, and evaporation) and nanoprecipitation (based on solvent displacement, where NPs form spontaneously through interfacial deposition of polymers). Additionally, methods such as self‐assembly, dialysis, and emulsion polymerization are widely applied in NP manufacturing [521].

In the design and synthesis of polymeric NPs, synthetic polymers are highly favored due to their controllable structures, ease of functionalization, and excellent batch‐to‐batch consistency. Precise molecular design enables efficient targeting and modulation of the tumor stroma. Amphiphilic block copolymers can self‐assemble into NPs with a core–shell structure, where the hydrophilic segment (e.g., polyethylene glycol, PEG) confers long‐circulating properties, and the hydrophobic segment (e.g., PLGA or poly(ε‐caprolactone) [PCL]) serves to encapsulate hydrophobic drugs [481, 522] Such NPs exhibit significant advantages in targeting the tumor stroma. For instance, mPEG–PLLys(BZD) nanovesicles (Gem/Sul‐NP) codeliver gemcitabine and the Pin1 inhibitor sulfopin, with a particle size of approximately 50 nm [475]. These NPs undergo pH‐responsive disassembly in the acidic TME (TME, pH 6.5), enabling controlled drug release. This system enhances chemotherapeutic efficacy by inhibiting pancreatic stellate cell activation and upregulating the gemcitabine transporter ENT1 on tumor cell membranes, synergizing with anti‐PD‐1 therapy to augment antitumor immune responses [475]. Another study employed mPEG–PLGA NPs loaded with baicalein (PMs‐Ba), which effectively reduced CAF activation by inhibiting TGF‐β/Smad and MAPK signaling pathways, thereby ameliorating the immunosuppressive TME in TNBC and enhancing the efficacy of DOX [473]. Furthermore, aminoethyl anisamide (AEAA)–PEG–PCL‐based targeted NPs codelivering silibinin and the PI3Kγ inhibitor IPI‐549 remodel the TME through synergistic modulation of CAFs and immunosuppressive cells, improving chemoimmunotherapy outcomes in breast cancer [476].

Dendritic polymers (e.g., polyamidoamine [PAMAM]; poly(oligo(ethylene glycol) methyl ether methacrylate) [POEGMA]) possess highly branched three‐dimensional structures and abundant terminal functional groups, facilitating multivalent modifications and multifunctional integration, making them ideal platforms for multistimuli‐responsive drug delivery [523, 524]. For example, POEGMA‐based dendritic nanomedicines (P‐DAS/P‐Epi) were designed for sequential therapy: P‐DAS conjugates the CAF metabolic modulator dasatinib (DAS) via a cathepsin B (CTSB)‐cleavable peptide (GFLG), enabling TME‐specific release of DAS to reprogram CAFs and reduce collagen deposition. P‐Epi links epirubicin (Epi) through a pH‐labile hydrazone bond, facilitating rapid drug release in the acidic TME to induce immunogenic cell death [474]. Another study utilized PAMAM dendrimers modified with N,N‐dipentylethylamine and mPEG to construct ultra‐pH‐sensitive NPs (SPN@Pro‐Gem). These particles maintain a size of approximately 120 nm in the bloodstream but rapidly disassemble into ∼8 nm particles in the acidic TME (pH ∼6.8), significantly promoting deep penetration of the gemcitabine prodrug into tumor parenchyma. This not only enhances chemotherapy but also creates conditions for a combination with ICI therapy by upregulating PD‐L1 level on tumor cells [20].

Semiconducting polymer NPs, beyond the advantages of traditional polymeric carriers, also exhibit functionalities such as photodynamic, photothermal, or sonodynamic effects, enabling the development of externally stimulus‐responsive intelligent delivery systems. For instance, Zhang et al. conjugated the LOX inhibitor BAPN to a semiconducting polymer via a CTSB‐cleavable peptide (GFLG), specifically inhibiting collagen cross‐linking in the TME to reduce ECM stiffness and promote immune cell infiltration [191]. Similarly, other studies reported conjugates of HPMA copolymers with collagen‐hybridizing peptides (CHPs), which specifically bind to denatured collagen chains in the tumor ECM, achieving tumor‐specific localization and prolonged retention of drugs, providing an effective delivery platform for ECM‐targeting therapeutics [525].·

Compared with synthetic polymers, natural polymer NPs demonstrate considerable potential in biomedical applications, particularly in stroma‐targeted drug delivery systems, owing to their superior biocompatibility, biodegradability, and inherent low toxicity [526, 527, 528]. However, unmodified natural polymers often suffer from drawbacks such as poor stability and insufficient targeting ability; thus, chemical modifications (e.g., introduction of hydrophobic groups, targeting ligands, or copolymerization with other polymers) are frequently required to optimize their performance [520].

Polysaccharide‐based polymers, such as chitosan, HA, and alginate, are naturally abundant and derived from renewable resources (e.g., crustaceans, connective tissues, and algae, respectively). They are also readily modifiable. Through hydrophobic modification or functional grafting, they can self‐assemble into stable NPs for targeted delivery [529]. For example, hydroxyethyl starch‐cholesterol (HES–CH) self‐assembled NPs codeliver the natural active product fucoxanthin (FX) and Twist‐targeting siRNA (siTwist/FX@HES–CH). These NPs, with a size of approximately 172 nm, effectively penetrate the tumor parenchyma of TNBC, synergistically inhibiting tumor cell proliferation, promoting apoptosis, and significantly reducing CAF activation and collagen deposition [526]. Another prominent example is the carboxymethylcellulose–DTX nano‐conjugate. This system is constructed by covalently linking acetylated carboxymethyl cellulose with PEG and DTX, yielding particles around 120 nm in size. It specifically targets and depletes CAFs in the TME of pancreatic cancer, thereby reducing stromal density and increasing tumor vascular perfusion [525]. Additionally, while semi‐synthetic, the conjugate of HPMA copolymer with CHP (HPMA–CHP) utilizes CHP, a targeting moiety derived from a natural source. This conjugate specifically recognizes and binds to denatured collagen in the tumor ECM, demonstrating superior tumor accumulation and longer retention compared with nontargeted polymers or free CHP in breast cancer models, offering a novel strategy for ECM‐targeted drug delivery [471].

Protein‐based polymers (e.g., gelatin, albumin) possess inherent amphiphilicity and drug‐binding sites, facilitating the loading of both hydrophobic and hydrophilic drugs. Albumin NPs (e.g., abraxane) are clinically used for tumor targeting, though their stroma specificity requires further design [530]. Moreover, polymer–protein hybrid NPs show great promise. For instance, pH‐responsive bovine serum albumin (BSA)–polymer core–shell NPs have been developed, featuring a drug‐loaded PLGA core surrounded by a cross‐linked BSA shell. This design minimizes serum protein adsorption and macrophage uptake, prolonging blood circulation time and enabling responsive drug release in the acidic TME, demonstrating potent anticancer activity and low toxicity to healthy tissues in both in vitro and in vivo studies [531, 532].

### 4.4 Carbon‐Based Nanomaterials

Carbon dots (CDs) have garnered significant attention due to their small size, excellent fluorescence properties, and good biocompatibility (Table 3). However, their relatively small dimensions, while facilitating favorable tissue penetration, also lead to short in vivo circulation times and rapid clearance [478, 533]. To address this limitation, researchers have assembled CDs into cluster structures or performed precise functionalization to significantly enhance drug targeting and delivery efficiency. For instance, Hou et al. constructed honeycomb‐like mesoporous nanoassemblies by crosslinking small‐sized CDs with FAP‐α‐sensitive peptide chains [481]. These materials disassemble in response to enzymatic cleavage within the tumor stroma, enabling the targeted release of LOS and the deep delivery of DOX/iron ions [481]. Another innovative strategy involves the construction of redox‐responsive CD nanoclusters (y‐CDCs). Guo et al. utilized disulfide bonds as reversible crosslinkers to assemble CDs into nanoclusters (y‐CDCs) of approximately 150 nm. In the TME with high glutathione (GSH) concentrations, the disulfide bonds break, causing the cluster structure to dissociate and revert to small‐sized CDs, thereby enhancing penetration. Further coating with cancer cell membrane (CCM) confers homologous targeting capabilities [478]. Additionally, directly conjugating hydrophobic drugs to CDs via covalent bonds demonstrates unique advantages. Mattinzoli et al. covalently linked the SN38 drug to the surface of CDs through an acid–labile carbamate bond, forming a CD‐SN38 complex. This linkage ensures drug stability during blood circulation (pH 7.4) and triggers controlled release in the acidic environment of the tumor cell nucleus (pH ∼5.3), significantly inhibiting the progression of CRC [482].

Graphene fluorescent NPs (GFNPs), with a size of approximately 3 nm and surfaces rich in functional groups such as –OH and SO^3^^−^, can achieve dual targeting of tumor cells and CAFs through electrostatic interactions and surface‐labeled proteins (e.g., α‐SMA). In pancreatic cancer models, the GFNPs–GEM system, formed by combining GFNPs with gemcitabine, exhibits good nuclear localization ability and penetration through fibrotic stroma [479].

Black phosphorus (BP)/carbon hybrid materials, formed by combining BP nanosheets with carbon‐based materials like CDs, significantly improve material stability and photothermal conversion efficiency [534]. For example, the NIR‐II‐CD/BP hybrid system exhibits excellent deep‐tissue penetration capability in the second NIR (NIR‐II) window. Its photothermal effect can induce thermal denaturation of collagen fibers in the ECM, reducing tissue stiffness and promoting the penetration and distribution of therapeutic agents [535]. Studies show that the photothermal conversion efficiency of this hybrid material under 808 nm and 1064 nm laser irradiation reaches 77.3 and 61.4%, respectively, significantly higher than that of pure BP nanosheets (49.5 and 28.4%), and it effectively inhibits tumor growth achieving complete ablation [536].

Furthermore, the LCTi carbon nanosystem, by loading LOS into hollow mesoporous carbon nanospheres (HMCNs) and modifying them with iRGD peptides for active targeting, achieves photothermal‐responsive controlled drug release [480]. Under 808 nm laser irradiation, the released LOS reprograms CAFs, thereby reversing the fibrotic state of the ECM [537]. In vivo experiments demonstrate that the LCTi system can achieve complete tumor eradication even in deep‐tissue tumor models (e.g., tumors covered with 10 mm thick tissue) and effectively suppress the formation of lung metastases [538].

### 4.5 Metal–Organic Frameworks

MOFs are extended porous network materials assembled via a bottom‐up building block approach through coordination bonds between metal‐based nodes (e.g., metal ions or clusters) and organic linkers [539]. MOFs are characterized by their high crystallinity, remarkable surface areas, and large pore volumes [540]. Owing to their modular construction and the chemical adaptability of their components (nodes and linkers), the structural topology, pore functionality, and crystal morphology of MOFs can be precisely tailored. In cancer therapy, MOFs can serve as nanoscale drug carriers, with key features including customizable pore dimensions (e.g., mesoporous structures), good biocompatibility (as certain MOFs can be synthesized under biocompatible conditions such as room temperature and aqueous environments), and the ability to achieve targeting capabilities through surface functionalization [541, 542, 543].

MOFs demonstrate significant advantages as drug carriers for targeting the tumor stroma, primarily owing to their high drug‐loading capacity, ease of functionalization, and favorable biocompatibility. The highly porous structure of MOFs enables the adsorption of a large quantity of drug molecules. For instance, the loading efficiency of DOX in zeolitic imidazolate framework 8 (ZIF‐8) can exceed 20 weight percent, ensuring efficient drug delivery capabilities [544]. Concurrently, the surface of MOFs can be modified with targeting ligands such as HA and folic acid, or with antibodies and peptides, to enhance specific recognition of tumor stromal components and improve targeting precision [545]. In terms of biosafety, Zr‐based MOFs (e.g., UiO‐66, MOF‐808) and Zn‐based MOFs (e.g., ZIF‐8) have exhibited low toxicity in both in vitro and in vivo experiments, providing a crucial foundation for their clinical translation [546].

Leveraging these advantages, MOFs have been widely applied to intervene in the tumor stroma to overcome chemoresistance and immunosuppression. For example, the Col–Afb–M808 system, formed by precoating MOF‐808 with collagenase, increased penetration depth by 50% in three‐dimensional tumor spheroids, significantly enhancing drug delivery efficiency to deep tumor regions [547]. Furthermore, MOF@siDDR2+siITGAV, by codelivering siRNAs targeting the ECM receptors DDR2 and ITGAV, reduced collagen deposition by 84.9% and promoted a threefold increase in CD8+ T cell infiltration in the 4T1 breast cancer model, effectively remodeling the immune microenvironment [548]. The MOF‐based nanoplatform OEMH NPs developed by Liu et al. delivered epigallocatechin gallate to inhibit the TGF‐β signaling pathway, blocking the transformation of normal fibroblasts into CAFs, reducing ECM stiffness, and thereby reversing oxaliplatin resistance [549].

However, MOF NPs face dual challenges of immune clearance and insufficient targeting specificity during in vivo circulation. Studies indicate that unmodified MOFs exhibit a short half‐life in the bloodstream and tend to accumulate in the mononuclear phagocyte system (e.g., liver and spleen), leading to off‐target toxicity [550, 551, 552]. To address this challenge, biomimetic coating technologies have emerged. For instance, the IZB@CCM platform utilizes a hybrid membrane derived from cancer cells and CAFs to encapsulate ZIF‐8, achieving dual targeting of both cancer cells and CAFs. This design significantly enhances the codelivery efficiency of the FAK inhibitor IN10018 and bismuth, resulting in a reduction of postradiotherapy tumor volume to 30% of the control group and a 60% decrease in p‐FAK expression in a cervical cancer model [550]. Similarly, ZIF‐8 camouflaged with an erythrocyte membrane mimics the surface properties of natural red blood cells, effectively prolonging blood circulation time and enhancing targeted accumulation at tumor sites [546]. Furthermore, poor controllability of drug release is a critical factor affecting therapeutic efficacy. Traditional MOFs relying on stimuli‐responsive release triggered by the TME (e.g., pH, GSH) often lack spatiotemporal precision [540, 544, 547, 553]. Studies have shown that ZIF‐8 relying solely on pH responsiveness may suffer from premature release or incomplete release in complex in vivo environments [545]. Consequently, exogenous stimulus‐responsive MOFs have been developed. For example, ZIF‐8‐based magnetic nanocomposites under an alternating magnetic field can achieve a drug release rate of up to 80%, concurrently reducing cell viability by 50%, enabling truly spatiotemporally controlled delivery [5]. Additionally, photothermal synergistic MOF platforms, regulated by NIR light, can precisely control the timing and location of drug release, significantly enhancing treatment precision [544].

## 5 Conclusions

Overall, the field of targeting the tumor stroma has achieved remarkable progress. At the basic research level, our understanding of the dynamic remodeling mechanisms of stromal components, their contribution to immunosuppression, and their role as physical barriers has been significantly deepened. Therapeutically, diverse strategies have emerged, ranging from direct degradation of stromal components and intervention in key signaling pathways to cell therapies and intelligent nanomaterials (Table 3) [451, 452]. Particularly important, the combination therapy of antiangiogenic drugs (e.g., Bevacizumab) with ICIs has demonstrated synergistic efficacy in various cancers, marking the transition of the “stromal normalization” strategy to enhance immunotherapy from concept to clinical practice [401, 402]. However, despite the considerable potential demonstrated in preclinical studies for enhancing drug delivery, overcoming therapeutic resistance, and inhibiting tumor progression by targeting the tumor stroma, its successful translation into broad and reliable clinical practice still faces severe challenges [35].

The tumor stroma is not a static barrier but a dynamic network undergoing continuous remodeling during tumorigenesis, progression, and under therapeutic pressure. This dynamic nature means that any static intervention strategy targeting a single stromal component may rapidly become ineffective. For instance, degrading the ECM (e.g., using PEGPH20) might improve drug penetration in the short term but could trigger compensatory feedback loops [67, 68]. More importantly, stromal cells, particularly CAFs and TAMs, exhibit high heterogeneity and plasticity. They encompass various functional subtypes with protumor and antitumor activities, yet current therapeutic strategies (e.g., targeting FAP to deplete CAFs) often lack subtype specificity [84, 85, 86]. Such “one‐size‐fits‐all” depletion strategies may inadvertently eliminate stromal cells with antitumor functions (e.g., apCAFs), thereby potentially attenuating therapeutic efficacy or even promoting tumor progression [90–94]. This functional duality (e.g., YAP signaling can promote immune evasion under certain conditions yet recruit CD8+ T cells in others) further complicates targeted therapy [46]. While the dense stroma physically impedes drug delivery, it also, to some extent, restricts tumor cell invasion and metastasis. Consequently, excessive or inappropriate disruption of the stromal structure may carry the risk of promoting tumor metastasis [156, 157]. For example, early attempts using collagenase to directly degrade collagen, while reducing IFP, might have “paved the way” for tumor cells [155]. This indicates that the ideal strategy should not be simply “destroying” the stroma but rather “normalizing” it [268, 269].

Significant differences exist in the composition, structure, and function of the stroma among different tumor types (e.g., pancreatic cancer, breast cancer, CRC) and even among different patients with the same tumor type [149, 150]. This inter‐tumoral and inter‐patient heterogeneity implies that a single stroma‐targeting regimen cannot be applicable to all scenarios. Currently, there is a lack of effective biomarkers capable of accurately predicting patient response to specific stroma‐targeted therapies. This directly contributed to the failure of several clinical trials; for instance, Pegvorhyaluronidase alfa (PEGPH20) failed to show overall survival benefit in unselected pancreatic cancer patients but might be effective in the subset of patients with high HA expression.

Targeting the tumor stroma represents a significant shift in the cancer treatment paradigm, with the ultimate goal of inhibiting the growth of the “seed” by reshaping the “soil” and enhancing the efficacy of existing therapies. Although currently facing multiple challenges including high heterogeneity of stromal components​ (e.g. diverse subtypes and functional states of CAFs), delivery barriers, target selectivity, and uncertainties in clinical translation efficacy, we are gradually charting a clearer path forward​ through the integration of interdisciplinary approaches such as precision medicine, advanced nanotechnology, the latest immunological discoveries, and artificial intelligence. Only by deeply understanding the complex ecology of the tumor stroma and developing intelligent, personalized intervention strategies that match it can we ultimately break through the impasse and translate the great potential of targeting the tumor stroma into tangible clinical benefits for cancer patients.

## Author Contributions

Furong Liu, Zhibin Liao, and Zhanguo Zhang conceived and revised the paper. Siwei Wang, Haofan Hu, and Weifeng Zeng drafted the manuscript. Siwei Wang prepared the figures. All authors discussed the contents and prepared the figures. Xiaoping Chen, Lu Qin, and Furong Liu helped revise the manuscript. All authors read and approved the final manuscript.

## Funding

This study was funded by National Natural Science Foundation of China (82303628, 82172976, 82573179) and Natural Science Foundation of Hubei Province, China (2024AFA076).

## Ethics Statement

The authors have nothing to report.

## Conflicts of Interest

The authors declare no conflicts of interest.

## Data Availability

Data sharing not applicable to this article as no datasets were generated or analyzed during the current study.
